# Coordination of the host Vps4-Vta1 complex and the viral core protein Ac93 facilitates entry of Autographa californica multiple nucleopolyhedrovirus budded virions

**DOI:** 10.1128/jvi.02182-24

**Published:** 2025-03-26

**Authors:** Xiaorong Yue, Ning Ji, Yixiang Ma, Qianlong Yu, Lisha Bai, Zhaofei Li

**Affiliations:** 1 State Key Laboratory of Crop Stress Biology for Arid Areas, Key Laboratory of Plant Protection Resources and Pest Management of Ministry of Education, Key Laboratory of Integrated Pest Management on the Loess Plateau of Ministry of Agriculture and Rural Affairs, College of Plant Protection, Northwest A&F University 546345 , Yangling, China; 2 Shandong Engineering Research Center for Environment-Friendly Agricultural Pest Management, College of Plant Health and Medicine, Qingdao Agricultural University 98431 , Qingdao, China; Wageningen University & Research, Wageningen, the Netherlands

**Keywords:** AcMNPV, Ac93, MIM1, Vps4-Vta1, *S. frugiperda*

## Abstract

The endosomal sorting complex required for transport (ESCRT) is a protein machine mediating membrane scission. In intraluminal vesicle (ILV) formation, ESCRT-0 targets cargoes and recruits ESCRT-I/-II to create membrane invagination, whereas ESCRT-III coordinates with the AAA ATPase Vps4 and its cofactor Vta1 to catalyze the membrane fission. Recently, ESCRT-I/-III and Vps4 were found to be involved in the entry of the baculovirus Autographa californica multiple nucleopolyhedrovirus (AcMNPV). However, the necessity of other ESCRT components and the interplay of viral proteins and ESCRTs in regulating the virus entry remain elusive. Here, we identified ESCRT-0 (Hse1 and Vps27), ESCRT-II (Vps22, Vps25, and Vps36), and Vta1 of *Spodoptera frugiperda*. RNAi depletion of Vta1 but not the components of ESCRT-0 or ESCRT-II in Sf9 cells significantly reduced budded virus (BV) production. Quantitative PCR together with confocal microscopy analyses indicated that Vta1 was required for internalization and endosomal trafficking of BV. In the late phase of infection, although Vps4 and Vta1 were both distributed to the nucleus and at the plasma membrane, depletion of Vta1 did not affect BV release. Further analysis revealed that 7 of 14 BV envelope proteins (Ac75, Ac93, E25, F-like, P33, P48, and vUbiquitin) interacted with Vps4 and Vta1. Intriguingly, Ac93 adopted a similar mode as ESCRT-III proteins to interact with the microtubule-interacting and transport (MIT) domains of Vps4 and Vta1 via its C-terminal MIT-interacting motifs (MIM1), and the interactions were necessary for BV internalization. Together, our studies highlight the coordination of Vps4-Vta1 and Ac93, and probably other BV envelope proteins, in facilitating entry of AcMNPV.

**IMPORTANCE:**

The endosomal sorting complex required for transport (ESCRT) system is involved in the entry of diverse DNA and RNA viruses. However, the interplay of viral proteins and ESCRTs in promoting virus endocytosis remains largely unknown. Here, we found that the ESCRT early acting factors ESCRT-0/-II were not necessary for infectious budded virus (BV) production of Autographa californica multiple nucleopolyhedrovirus (AcMNPV). In contrast, the Vps4 cofactor Vta1 was required for entry but not egress of BV. Several core or essential BV envelope proteins were identified to interact with Vps4 and Vta1. Among them, Ac93 plays a central role in connecting other viral proteins and mimics ESCRT-III proteins to interact with Vps4-Vta1, facilitating entry of BV virions. These studies provide evidence for the coordination of viral proteins and ESCRTs in regulating entry of large enveloped DNA viruses.

## INTRODUCTION

From archaea to mammals, the endosomal sorting complex required for transport (ESCRT) is a conserved protein machine driving membrane deformation and fission (1, 2). The core machinery comprises Bro1-domain proteins (ALIX and HD-PTP), ESCRT-I, ESCRT-II, ESCRT-III, and the AAA ATPase Vps4 and its cofactor Vta1 (LIP5 in mammals) (2, 3). This complex plays essential roles in diverse membrane remodeling processes that include biogenesis of multivesicular bodies (MVBs), cytokinetic abscission, membrane repair, and virus exit from host cells (1).

For its canonical role in mediating yeast endosomal sorting and intraluminal vesicle (ILV) formation, the ESCRT activity is initiated as Hse1-Vps27 (known as ESCRT-0) binds to the membrane of endosomes and captures internalized ubiquitylated cargoes. ESCRT-0 recruits ESCRT-I (composed of Vps23/Tsg101, Vps28, Vps37, and MVB12/UBP1) and hands over the cargo. ESCRT-I recruits ESCRT-II (Vps22, Vps25, and Vps36) and, together, they create an invagination of the endosome membrane into which the cargo is sorted. Then, ESCRT-II recruits ESCRT-III subunits (Ist1, Vps2, Vps20, Vps24, Snf7, Vps46, and Vps60) to form the ESCRT-III filament, which constricts the membrane bud neck. Following the membrane deformation, Vps4 and its cofactor Vta1 are recruited through the interaction of the microtubule-interacting and transport (MIT) domains of Vps4 and Vta1 with the MIT-interacting motifs (MIMs) of ESCRT-III subunits (1, 2). Finally, the Vps4-Vta1 complex hydrolyzes ATP to promote membrane scission and disassemble the ESCRT-III complex (Fig. 9, left panel) (4, 5).

In viral infection, there is substantial evidence that viruses hijack the ESCRT machine to facilitate their propagation (1, 6). In established models, it is well understood that retroviruses, particularly human immunodeficiency virus type 1 (HIV-1), utilize their Gag proteins as the adaptor to recruit ALIX, NEDD4, and/or Tsg101, which in turn recruit ESCRT-III and Vps4 to promote viral budding (1, 7). Recently, an increasing number of studies have demonstrated that ESCRTs play vital roles in the entry of various viruses, including the arenaviruses (lassa virus, LASV; lympholytic choriomeningitis virus, LCMV) (8), coronaviruses (porcine enteric alphacoronavirus, porcine epidemic diarrhea virus) (9), baculovirus (Autographa californica multiple nucleopolyhedrovirus, AcMNPV) (10–12), flavivirus (classical swine fever virus, CSFV) (13), herpesvirus (kaposi’s sarcoma-associated herpesvirus, KSHV) (14, 15), nairovirus (Crimean-Congo hemorrhagic fever virus) (16), papillomavirus (human papillomavirus, HPV) (17), piconavirus (echovirus 1) (18), and rotavirus (rhesus rotavirus, RRV) (19). Although the non-structural proteins of CSFV and capsid proteins of HPV have been determined to interact with Tsg101 or Vps4, the coordination of viral proteins and ESCRTs in regulating virus entry remains elusive.

Baculoviruses are large enveloped dsDNA viruses known to infect insects (20). AcMNPV is the most extensively studied baculovirus that serves as a powerful system for expressing proteins, producing vaccines, and transducing mammalian cells (21–23). During infection, the budded form of AcMNPV (budded virus, BV) binds to an unknown receptor at the cell surface and enters host cells via clathrin-mediated endocytosis (CME) (20, 24). In endosomes, low-pH triggered conformational change of GP64, a class III fusogen, mediates fusion of the viral envelope and endosomal membranes to release nucleocapsids into the cytoplasm (25, 26). Then, the nucleocapsids are propelled by actin filaments and transported to the nucleus for replication (27). After assembly, one set of nucleocapsids exits the nuclei and buds from the plasma membrane to form BV, while the others remaining in the nucleus are wrapped by intranuclear microvesicles (IMs) to form occlusion-derived viruses (ODVs) (20). Although both phenotypes of the virus are generated in the same infected cell, BV rather than ODV can establish efficient infection in cell culture (20). Recent studies revealed that the ESCRT core complexes ESCRT-I/-III and Vps4 of *Spodoptera frugiperda* are involved in entry and egress of AcMNPV (10–12). A number of BV-related proteins, including the envelope and nucleocapsid proteins and non-structural proteins required for BV production, form a complicated interaction network with ESCRT-III subunits (11). Among them, the viral core protein Ac93 shares a conserved MIM1 motif with the ESCRT-III proteins. Mutations of conserved leucine residues in MIM1 disrupt the association of Ac93 with ESCRT-III/Vps4 and impair BV egress from the nuclear envelope (NE) (28). In addition, overexpression of truncated Vta1 lacking the N-terminal MIT domains affects BV production (29). These accumulated data suggest that AcMNPV usurps ESCRTs to propagate the infection, but the mechanisms are not clear.

In this study, we identified the components of ESCRT-0 (Vps27 and Hse1) and ESCRT-II (Vps22, Vps25, and Vps36) of *S. frugiperda* and assessed the roles of these ESCRT early acting factors and Vta1 in AcMNPV infection. After determining the requirement of Vta1 but not ESCRT-0/-II for efficient BV entry, we mapped the interaction of Vps4-Vta1 and 14 BV envelope proteins. Based on the strong interaction of Ac93 and Vps4-Vta1, we further exploited the interaction mechanism and its necessity for BV entry.

## RESULTS

### Characterization of ESCRT-0/-II and Vta1 of *S. frugiperda*

**Fig 1:**
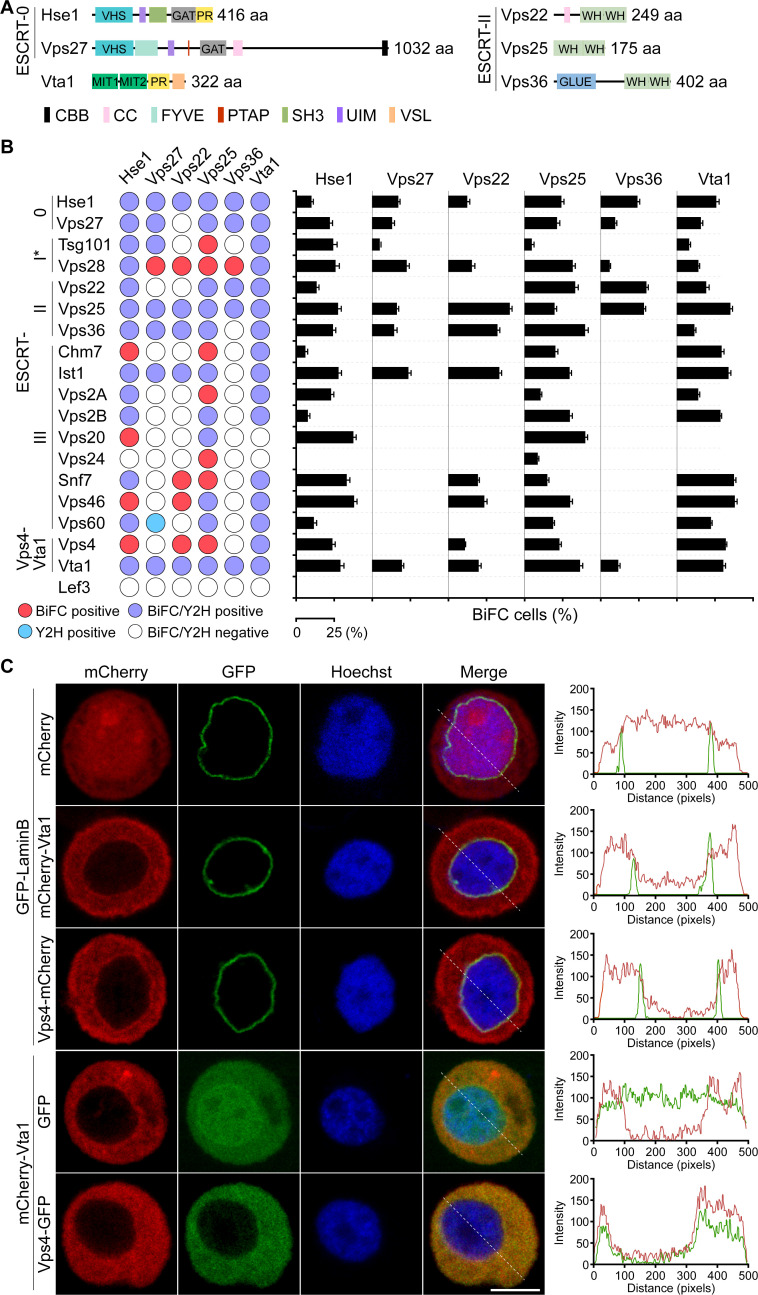
Characterization of ESCRT-0/-II and Vta1 of *S. frugiperda*. (A) Domain architectures of the components of ESCRT-0/-II and Vta1. CBB, clathrin-binding box; CC, coils coil; FYVE, Fab-1/YGL023/Vps27/EEA1; GAT, GGA and Tom1; GLUE, GRAM-like ubiquitin-binding in EAP45; MIT, microtubule-interacting and transport; PR, proline-rich; SH3, SRC homology 3; UIM, ubiquitin-interacting motif; VHS, Vps27/Hrs/STAM; VSL, Vta1/SBP1/LIP5; WH, winged helix-like DNA-binding domain. (B) Y2H and BiFC analyses of the interactions of the components of ESCRT-0/-II and Vta1 with other ESCRT proteins. Lef3 of AcMNPV was used as a negative control. The BiFC of Vta1 and Vps46 or Vps60 identified in a prior study (29) were used as the positive control. Note: the ESCRT-I components Vps37 and MVB12 were not included in these assays. Error bars represent the standard deviation (SD) from the mean of three replicates. (C) Colocalization analysis of mCherry-tagged Vps4 and Vta1 with GFP-LaminB, and mCherry-Vta1 and Vps4-GFP in transfected Sf9 cells. Plots of pixel intensity along the white dashed line are shown on the right side of the images. Scale bar, 10 μm.

Sequence alignments showed that the components of ESCRT-0 (Hse1 and Vps27), ESCRT-II (Vps22, Vps25, and Vps36), and Vta1 of *S. frugiperda* share about 21%–35%, 45%–50%, and 50%–90% amino acid identities with the homologs of yeast, humans, and other insects, respectively. For Hse1 and Vps27, their N-terminal regions (about 380 aa in Hse1 and 500 aa in Vps27) are highly conserved and mainly composed of VHS (Vps27/Hrs/STAM domain), UIM (ubiquitin-interacting motif), GAT (GGA and Tom1) (Hse1, Vps27), SH3 (SRC homology 3 domain) (Hse1), and FYVE (Fab-1/YGL023/Vps27/EEA1) (Vps27) domains. In contrast, the C-termini of their homologs are variable in length. In addition, the C-terminal region (365–416 aa) of *S. frugiperda* Hse1 is rich in proline, while that of Vps27 (471–1019 aa) is rich in methionine and glutamine (Fig. 1A, extended Fig. S1 and S2). Similar to the orthologs of other eukaryotes, the ESCRT-II components Vps22, Vps25, and Vps36 of *S. frugiperda* contain two tandem winged helix-like DNA-binding domains (WH). In addition, Vps22 and Vps36 contain a coils coil (CC) or a GLUE (GRAM-like ubiquitin-binding in EAP45) domain at the N-terminal region. The Vps4 cofactor Vta1 is also highly conserved and contains two MIT domains, a proline-rich region, and the C-terminal VSL (Vta1/SBP1/LIP5) motif (Fig. 1A). Yeast two-hybrid (Y2H) and bimolecular fluorescence complementation (BiFC) analyses showed that Hse1, Vps25, and Vta1 broadly interacted with the other ESCRT proteins, while Vps22 and Vps27 interacted with one or a few components of ESCRT-0, -I, -II, -III, or Vps4-Vta1. For Vps36, the interaction only occurred between it and the components of ESCRT-0 (Hse1, Vps27), ESCRT-I (Vps28), ESCRT-II (Vps22 and Vps25), or Vta1 (Fig. 1B, left panel). In general, the percentage of fluorescent cells in BiFC was about 10%–40%. However, those for the interaction between Hse1 and Chm7 or Vps2B, Vps25 or Vps27 and Tsg101, or Vps28 and Vps36 were only about 5%, suggesting weak associations of these ESCRT components (Fig. 1B, right panel).

To determine the subcellular localization of Vta1, mCherry was fused to its N-terminus, and the fusion construct was transiently expressed in Sf9 cells. Confocal microscopy indicated that mCherry-Vta1 was evenly distributed in the cytoplasm, and a portion of the fusion protein was colocalized with the GFP-tagged NE marker protein LaminB. This localization pattern was similar to that of mCherry-tagged Vps4. In cotransfected Sf9 cells, mCherry-Vta1 was nearly completely colocalized with Vps4-GFP (Fig. 1C).

### Vta1 but not ESCRT-0/-II is required for infectious BV production

**Fig 2:**
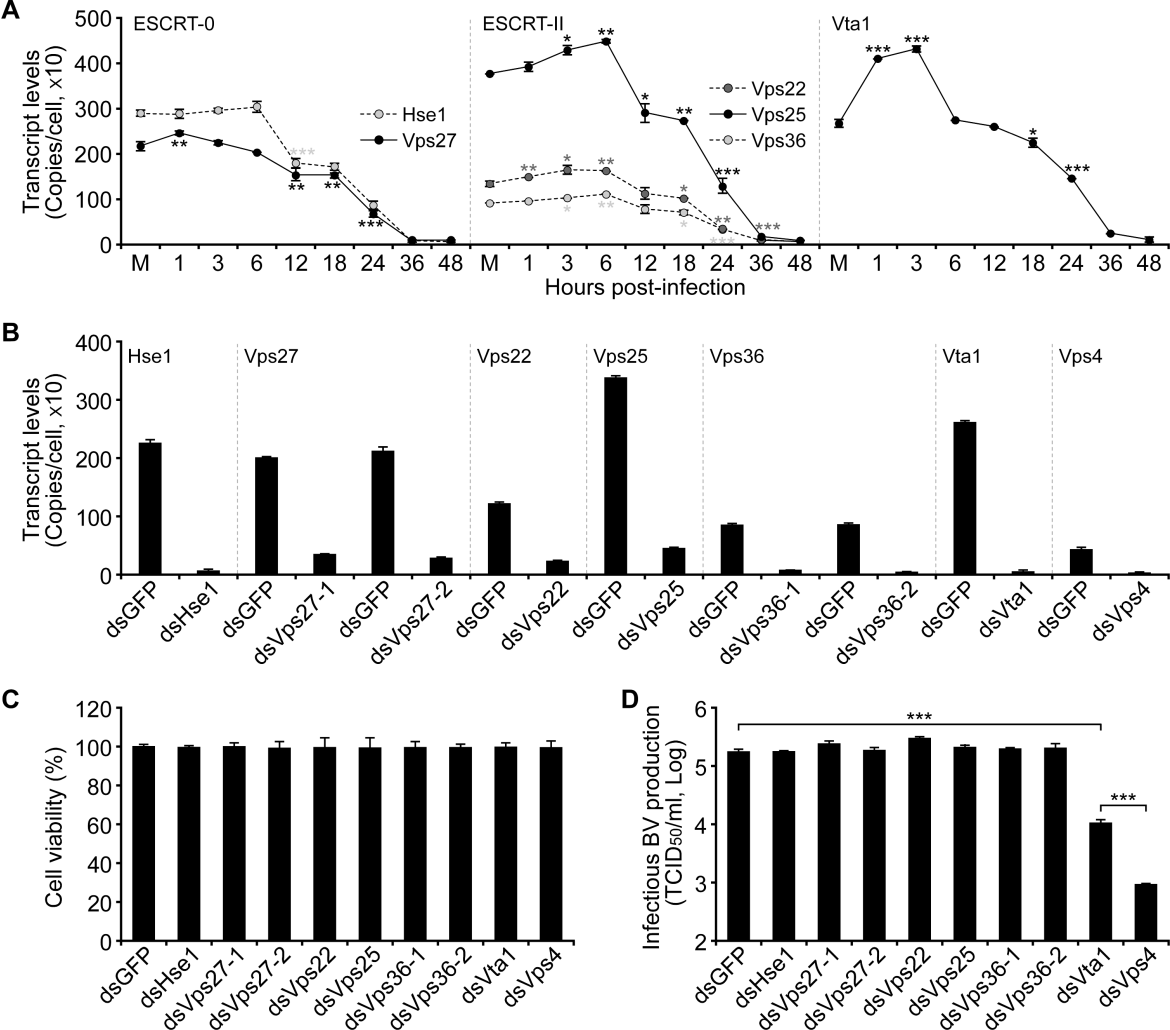
Effects of depletion of ESCRT-0/-II components or Vta1 on infectious BV production. (A) Transcriptional changes of the components of ESCRT-0/-II and *Vta1* upon AcMNPV infection (MOI = 5). M, mock-infected cells. (B–D) Effects of RNAi on transcript levels of each gene (B), the viability of Sf9 cells (C), and infectious BV production (D). After transfecting the dsRNA for 48 h, the cells were infected with AcMNPV-LacZGUS (MOI = 5). At 24 h p.i., the infectious BVs were titered by a TCID_50_ assay. Error bars represent SD from the mean of three replicates. *, *P* < 0.05; **, *P* < 0.01; ***, *P* < 0.001 (by paired two-tailed *t* test).

To ask whether ESCRT-0/-II and Vta1 are necessary for AcMNPV infection, we first measured the transcript levels of these components in infected Sf9 cells. During the early stage of infection (1–6 h post-infection [p.i.]), except for that of *Hse1* changing slightly*,* the transcript levels of other components were significantly up-regulated at 1 h p.i. (*Vps27*), 1–3 h p.i. (*Vta1*), 1–6 h p.i. (*Vps22*), or 3–6 h p.i. (*Vps25* and *Vps36*). After 6 h p.i., the transcript levels for each gene were decreased, and at 36–48 h p.i., they remained only about 1%–5% (Fig. 2A).

To determine the requirement of these ESCRT components in AcMNPV infection, dsRNA-based RNAi was used to assess the effects of depletion of their transcripts on infectious BV production. At 48 h post-transfection (p.t.), transfection of the dsRNA targeting each component of ESCRT-0, ESCRT-II, or *Vta1* significantly knocked down the transcript levels over 90%–95% but did not substantially alter the viability of Sf9 cells (Fig. 2B and C). In comparison with the control dsRNA of *gfp*, depletion of the components of ESCRT-0 or ESCRT-II had no significant impact on BV production. However, depletion of *Vta1* remarkably reduced the production of infectious BV (about 30-fold), and a more severe reduction of the virus titer was detected for depletion of *Vps4* as previously described (12) (Fig. 2D). These results suggested that Vta1, but not ESCRT-0/-II, is required for producing infectious BV.

### Vps4-Vta1 is involved in internalization and endosomal trafficking of BV during entry

**Fig 3:**
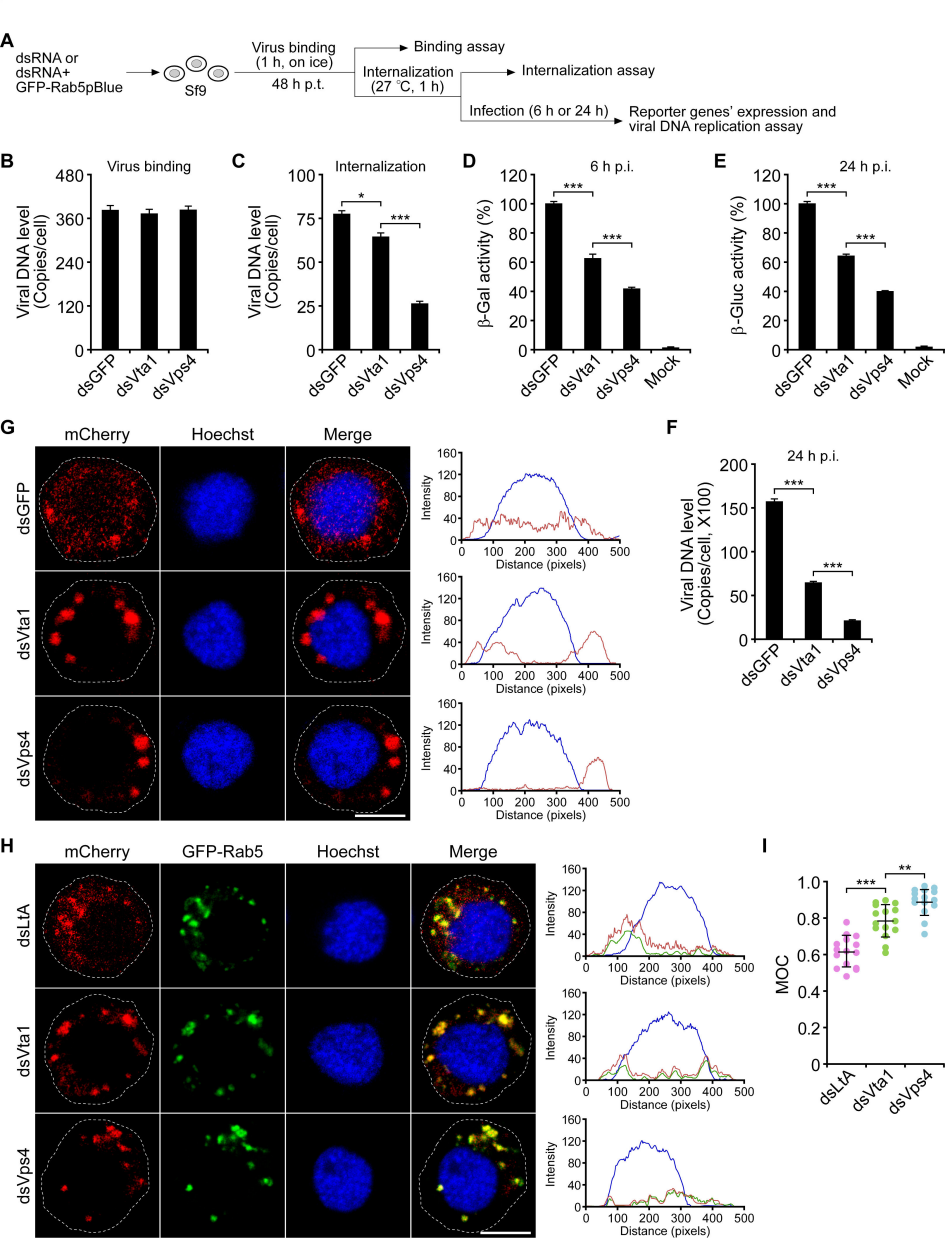
Effects of depletion of Vps4 or Vta1 on BV binding and internalization. (A) Schematic representation of the assay. (B–F) Sf9 cells were transfected with the dsRNA of *gfp*, *Vps4*, or *Vta1*. At 48 h p.t., the cells were inoculated with the purified AcMNPV-LacZGUS (MOI = 10), and the virus binding and internalization were determined by assessing viral DNA using qPCR (B, C). At 6 h and 24 h p.i., the cells were lysed to measure the activities of β-Gal (D) and β-Gluc (E), and the amount of viral DNA (F). Error bars represent SD from the mean of three replicates. *, *P* < 0.05; ***, *P* < 0.001 (by paired two-tailed *t* test). (G–I) Sf9 cells were transfected with the dsRNA of *gfp*, *Vps4*, or *Vta1*, or cotransfected with a plasmid expressing GFP-Rab5 and the dsRNA of *LtA*, *Vps4*, or *Vta1*. At 48 h p.t., the cells were inoculated with AcMNPV-3mC (MOI = 20). After binding on ice for 1 h, the virus was internalized at 27°C for 1 h. Then, the cells were processed to be imaged by confocal microscopy (G, H). Plots of pixel intensity of the whole cell are shown on the right side of the confocal images. Colocalization of internalized virions and GFP-Rab5 was evaluated by calculating the Manders overlap coefficient (MOC) of 15 representative cells for each treatment (I). **, *P* < 0.01; ***, *P* < 0.001 (by paired two-tailed *t* test). Scale bar, 10 µm.

Since entered BV virions are mainly transported to early and maturing endosomes to release nucleocapsids (30, 31), the negative effect of Vta1 depletion on BV production may be related to impaired virus entry. To test this, Sf9 cells were initially transfected with the dsRNA of *gfp*, *Vps4*, or *Vta1*, and then inoculated with the virus AcMNPV-LacZGUS that encodes β-galactosidase (β-Gal) and β-glucuronidase (β-Gluc) under the control of AcMNPV *ie1* early promoter and *p6.9* late promoter, respectively (Fig. 3A). Quantitative PCR (qPCR) analysis showed that the amount of BV virions attached to the cells was similar in cells transfected with the dsRNA of *gfp*, *Vps4*, or *Vta1,* suggesting that depletion of *Vps4* or *Vta1* had no significant effect on the virus binding (Fig. 3B). However, depletion of *Vps4* or *Vta1* substantially reduced the quantity of BV virions internalized into Sf9 cells (Fig. 3C). At 6 h and 24 h p.i., the activities of β-Gal and β-Gluc in Sf9 cells transfected with the dsRNA of *Vps4* or *Vta1* were significantly decreased about 58%–60% (*Vps4*) and 35%–37% (*Vta1*) in comparison with that of dsGFP (Fig. 3D and E). Consistent with the defect of reporter genes’ expression, at 24 h p.i., the dsRNA treatment also caused a significant reduction of the amount of viral genomic DNA (about 2.4-fold to 7.2-fold) (Fig. 3F).

To track BV internalization and trafficking, Sf9 cells were transfected with the dsRNA of *gfp*, *Vps4*, or *Vta1* and then inoculated with the virus AcMNPV-3mC, which contains the 3×mCherry-tagged major capsid protein VP39 (27). Confocal microscopy showed that internalized virions were distributed uniformly in the cytoplasm in Sf9 cells transfected with the dsRNA of *gfp*, and a certain amount of mCherry-labeled nucleocapsids were observed in the nucleus. In contrast, similar to that in cells transfected with the dsRNA of *Vps4*, entered virions were mostly aggregated in the cytoplasm of cells transfected with the dsRNA of *Vta1* (Fig. 3G). To further determine the subcellular localization of internalized virions, Sf9 cells were cotransfected with the dsRNA of *Mus musculus lymphotoxin A* (*LtA*) (note: LtA has no homologs in insects and was used as a negative control as described previously [32]), *Vps4*, or *Vta1* and the plasmid GFP-Rab5pBlue, and then inoculated with the virus AcMNPV-3mC. Confocal microscopy revealed that mCherry-labeled virions in the cytoplasm were mainly colocalized with the GFP-tagged Rab5, which serves as the marker protein of early and maturing endosomes (Fig. 3H, the intensity plots). Manders overlap coefficient (MOC) measurements indicated that about 88.6% or 78.6% of the mCherry intensities in cells transfected with the dsRNA of *Vps4* or *Vta1* were colocalized with that of GFP-Rab5, and they were much higher than that in cells transfected with the dsRNA of *LtA* (Fig. 3I), suggesting that the internalized virions mainly localized within early and maturing endosomes. Taken together, these results indicated that the Vps4-Vta1 complex is required for efficient internalization and endosomal trafficking of BV during entry.

### Vta1 is not necessary for infectious BV release

**Fig 4:**
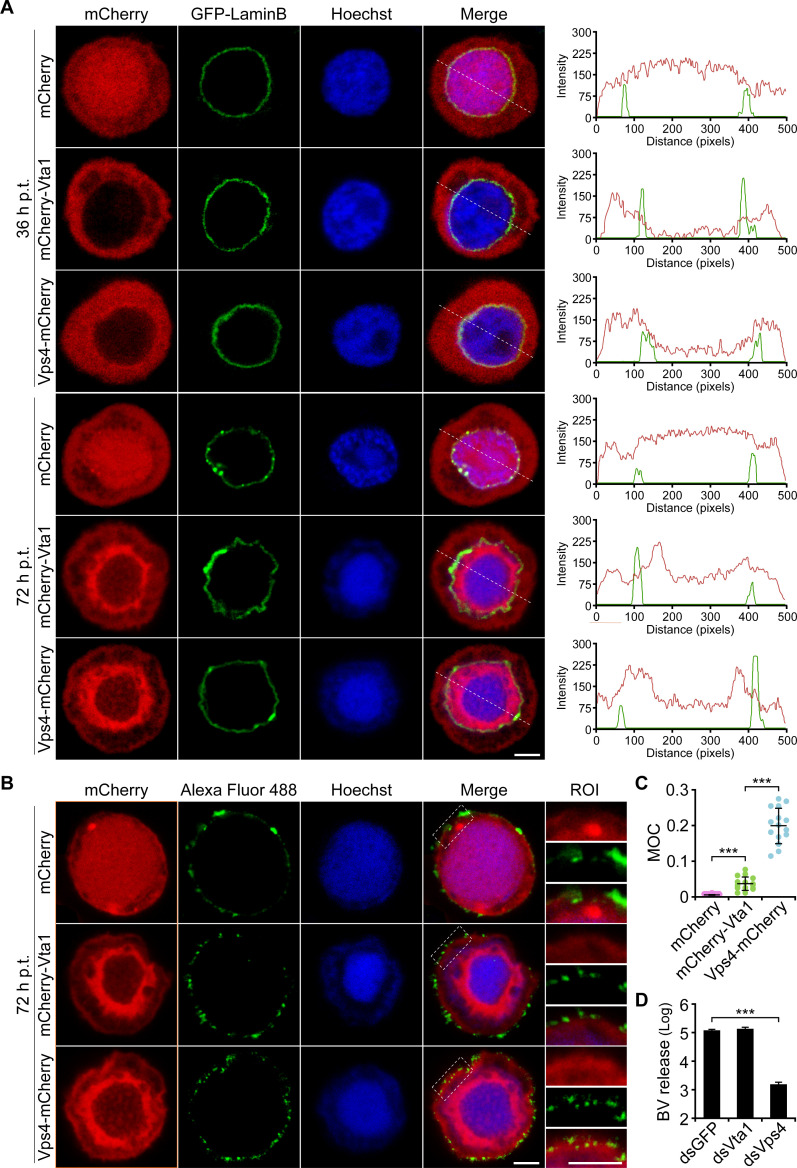
Distribution of Vps4 and Vta1 in infected cells and effects of Vta1 depletion on BV release. (A) Subcellular localization of mCherry-Vta1 and Vps4-mCherry in infected cells. The cells were cotransfected with GFP-LaminBpBlue and the bacmid expressing mCherry or mCherry-tagged Vps4 or Vta1 and imaged by confocal microscopy. Plots of pixel intensity along the white dashed line are shown on the right side of the images. Scale bar, 5 μm. (B) Colocalization of mCherry-tagged Vps4 or Vta1 and GP64 at the plasma membrane. Sf9 cells were transfected with the bacmid expressing mCherry or mCherry-tagged Vps4 or Vta1. At 72 h p.t., the cells were processed for immunofluorescence analysis. A zoomed view of the boxed regions of interest is shown on the right side of each panel. Colocalization was assessed by measuring MOC of 15 representative cells for each construct (C). Scale bar, 5 µm. (D) Effects of Vta1-depletion on BV release. Sf9 cells were transfected with the dsRNA of *gfp*, *Vps4*, or *Vta1*. At 48 h p.t., the cells were transfected again with the bacmid of AcMNPV-LacZGUS for 24 h. Then, the BV production was determined by a TCID_50_ assay. Error bars represent SD from the mean of three replicates. ***, *P* < 0.001 (by paired two-tailed *t* test).

Since Vps4 is also required for BV egress (10), the defect of BV production in Sf9 cells transfected with the dsRNA of *Vta1* may also result from the impaired BV release. To test this, we first cotransfected Sf9 cells with GFP-LaminBpBlue and the bacmid expressing mCherry or mCherry-tagged Vps4 or Vta1 and examined the subcellular localization of the two proteins at early and late stages of infection. Confocal microscopy showed that, similar to those observed in plasmid-transfected cells (Fig. 1C), at 36 h p.t., mCherry-Vta1 or Vps4-mCherry was distributed uniformly in the cytoplasm and on NE. At 72 h p.t., the fused proteins were mainly accumulated to the ring zone at the periphery of the nucleus, with a certain amount of the proteins closely distributed to the plasma membrane (Fig. 4A). To further assess if the mCherry-tagged proteins were localized to the plasma membrane, Sf9 cells were transfected with the bacmid expressing mCherry, mCherry-Vta1, or Vps4-mCherry. At 72 h p.t., immunofluorescence analysis revealed that a certain portion of mCherry-Vta1 or Vps4-mCherry was colocalized with the viral major envelope protein GP64 at partial regions of the plasma membrane (Fig. 4B). MOC measurements indicated that about 19.9% cell surface-localized GP64 was colocalized with Vps4-mCherry, while only 3.6% of the GP64 was colocalized with mCherry-Vta1 (Fig. 4C).

To assess the requirement of Vta1 for infectious BV release, Sf9 cells were transfected with the dsRNA of *gfp*, *Vps4*, or *Vta1*, and at 48 h p.t., the cells were transfected again with the bacmid of AcMNPV-LacZGUS. At 24 h p.t., the activities of β-Gal and β-Gluc in each set of the cells were similar (extended Fig. S3), suggesting that the virus infection cycle progressed into the late stage. As described in prior studies (12), knockdown of *Vps4* significantly reduced the amount of infectious BV. In contrast, depletion of *Vta1* had no negative influence on BV production (Fig. 4D). Taken together, these results indicated that Vps4 and Vta1 were distributed with a similar pattern in infected Sf9 cells. However, the much lower quantity of Vta1 localized on the plasma membrane may correlate with its unnecessary role in BV release.

### Interaction of Vps4-Vta1 and BV envelope proteins

**Fig 5:**
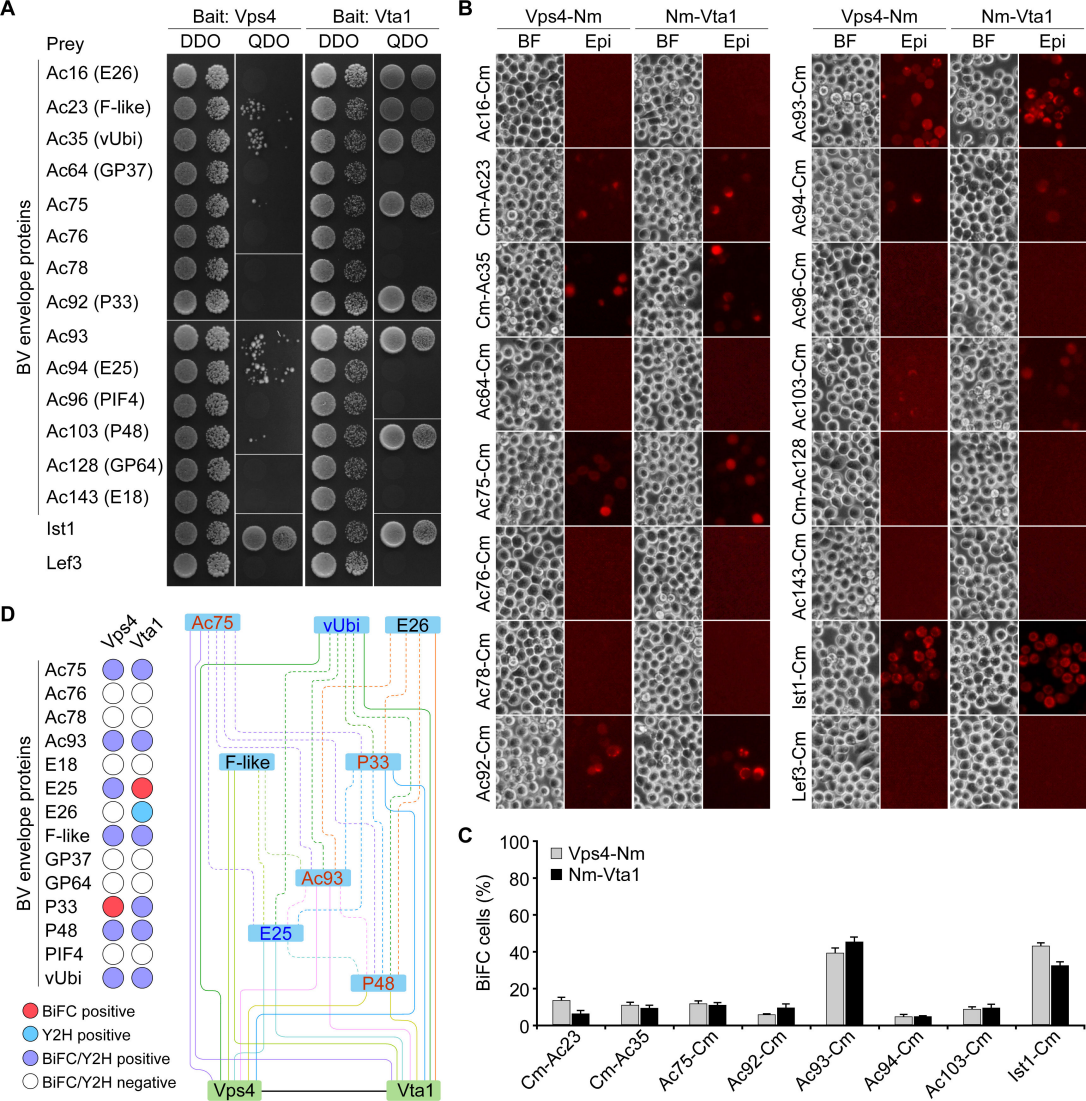
Y2H and BiFC analyses of the interaction between Vps4 or Vta1 and BV-envelope proteins of AcMNPV. (A) Y2H assay. A 10-fold dilution of yeast cells was plated. DDO, SD/-Leu/-Trp; QDO, SD/-Ade/-His/-Leu/-Trp. (B and C) BiFC assay. For comparison, the BiFC analysis of the interaction between Vps4 and Ac76, Ac78, Ac93, or Ac103 in a prior study (11) was re-analyzed. Nm and Cm represent the N- and C-terminal domain of mCherry, respectively. BF, bright field; Epi, epifluorescence. Error bars represent SD from the mean of three replicates. Ist1 and Lef3 were used as the positive and negative controls. (D) Summary of the interactions of Vps4-Vta1 with BV envelope proteins. The interactions identified in prior studies (11) were indicated as dashed color lines. The names of essential and necessary envelope proteins for AcMNPV infection were marked as red and blue, respectively.

AcMNPV enters host cells via CME (24). In endosomes, the conformational change of GP64 promotes the fusion of BV envelope and endosomal membranes to release nucleocapsids into the cytosol (25, 26). Requirement of Vps4-Vta1 for ILV formation and BV entry suggests that this complex might interact with BV envelope proteins to regulate the pathological process. To examine this possibility, we first performed the Y2H screen using Vps4 or Vta1 as the bait and 14 BV envelope proteins of AcMNPV, including Ac16 (E26), Ac23 (F-like), Ac35 (vUbi), Ac64 (GP37), Ac75, Ac76, Ac78, Ac92 (P33), Ac93, Ac94 (E25), Ac96 (PIF4), Ac103 (P48), Ac128 (GP64), and Ac143 (E18) (11) as the prey. In cotransformed yeast cells, five viral proteins including Ac75, Ac93, F-like, P48, and vUbi were found to interact with Vps4 and Vta1. In addition, E25 was found to interact with Vps4, and E26 and P33 interacted with Vta1 (Fig. 5A). Similar interactions were detected by swapping the viral proteins as the bait and Vps4 or Vta1 as the prey (extended Fig. S4).

To verify the interactions detected in the Y2H assay, we further performed the BiFC analysis in living Sf9 cells. The cells were cotransfected with a pair of BiFC plasmids separately expressing Vps4 or Vta1 fused with the N- or C-terminal domain of mCherry (Nm, Cm) and a viral protein fused with Cm or Nm. Western blotting indicated that all the fusion constructs were expressed in transfected cells (extended Fig. S5). Similar to that observed in Y2H analysis, the positive interactions between Vps4 or Vta1 and Ac75, Ac93, E25, F-like, P33, P48, or vUbi were also detected as positive in the BiFC assay. In addition, the negative interactions between Vps4 and P33, and Vta1 and E25 in Y2H assay were also observed as positive in BiFC analysis (Fig. 5B). Generally, the percentage of BiFC fluorescent cells ranged from 6.1% to 13.3% for Vps4 or Vta1 and most of the viral proteins. However, Ac93 showed 39.1% and 45.2% complemented fluorescent cells with Vps4 and Vta1, respectively (Fig. 5C). Combined with the Y2H and BiFC analyses, 7 BV envelope proteins (Ac75, Ac93, E25, F-like, P33, P48, and vUbi) were found to interact with Vps4 and Vta1. In addition, Vta1 might also interact with E26. Based on the identified interactions and the complex association of BV envelope proteins (11), an interaction network of BV envelope proteins and Vps4-Vta1 was established (Fig. 5D). In the network, Ac93 plays the predominant role in connecting the other envelope proteins and the Vps4-Vta1 complex.

### Vps4 and Vta1 interact with the MIM1 motif of Ac93

**Fig 6:**
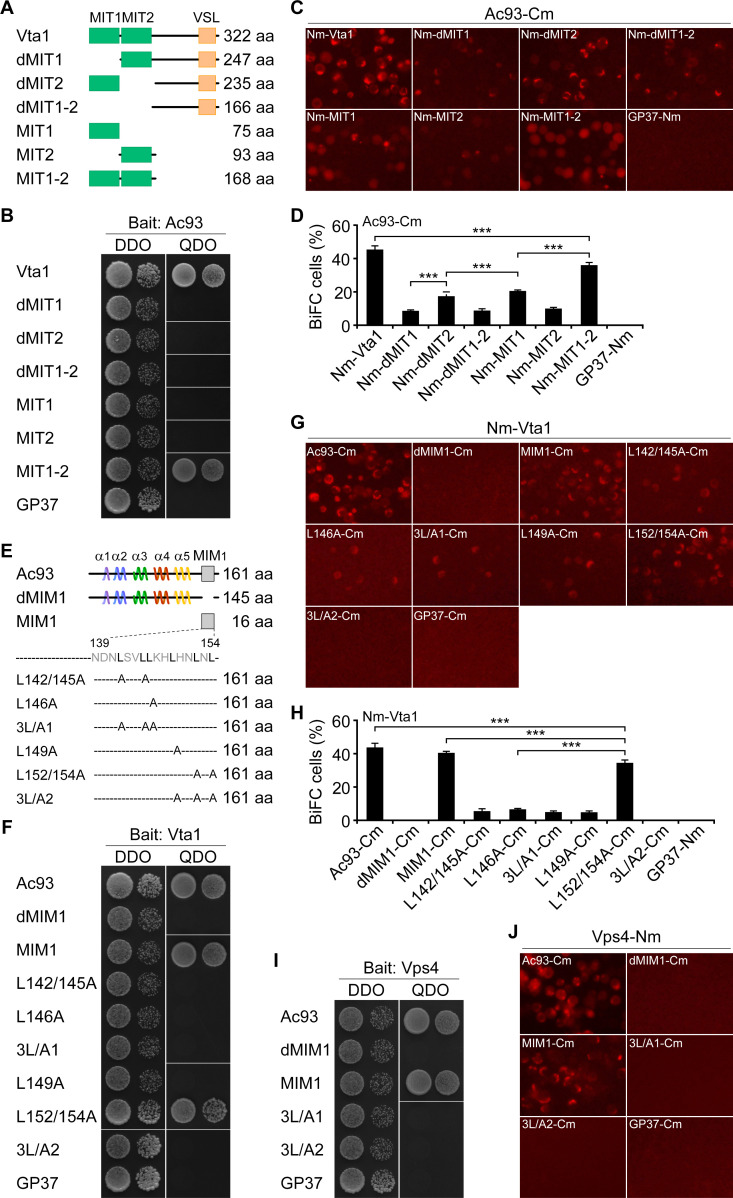
Y2H and BiFC analyses of the interaction of Ac93 with Vps4 and Vta1. (A) Schematic diagram of Vta1 constructs. (B–D) Y2H and BiFC analyses of the interaction of Ac93 with wt and truncated Vta1. In the Y2H assay, a 10-fold dilution of yeast cells was plated. DDO, SD/-Leu/-Trp; QDO, SD/-Ade/-His/-Leu/-Trp. (E) Schematic diagram of Ac93 constructs. (F–J) Y2H and BiFC analyses of the interaction of wt or modified Ac93 with Vta1 and Vps4. A 10-fold dilution of yeast cells was plated. DDO, SD/-Leu/-Trp; QDO, SD/-Ade/-His/-Leu/-Trp. GP37 was used as a negative control. Error bars represent SD from the mean of three replicates. ***, *P* < 0.001 (by paired two-tailed *t* test). MIM1, type I MIT-interacting motif.

Similar to ESCRT-III proteins, Ac93 is composed of five alpha helices and a C-terminal MIM1 motif (28). To gain insight into the interaction mechanism of Vps4-Vta1 and Ac93, we first constructed the truncated Vta1, including dMIT1 (deletion of the N-terminal MIT1 domain), dMIT2 (deletion of MIT2), dMIT1-2 (deletion of MIT1 and MIT2), MIT1, MIT2, and MIT1-2 (Fig. 6A), and analyzed their interactions with Ac93. Y2H analysis showed that the positive interaction was detected only for the construct MIT1-2. Deletion of MIT1 and/or MIT2 (dMIT1, dMIT2, and dMIT1-2), or expression of MIT1 or MIT2 abolished the interaction of the modified Vta1 and Ac93 (Fig. 6B). Further BiFC assay indicated that, in transfected Sf9 cells, all of the Nm-fused Vta1 truncations formed the complemented fluorescence with Ac93-Cm (Fig. 6C). However, in comparison with that of wild-type (wt) Vta1 (44.3%), the percentage of fluorescent cells for the modified Vta1 was significantly decreased (Fig. 6D). Among them, MIT1-2 was detected with many more fluorescent cells (34.6%) than that for MIT1 or dMIT2 (16.9% or 19.7%), and dMIT1, dMIT1-2, and MIT2 showed similarly low levels of fluorescent cells (8.5%–9.9%). These results indicated that MIT1 plays a major role in mediating the interaction of Vta1 and Ac93.

In prior studies, deletion of the Vps4 MIT domain or substitution of leucine residues within the MIM1 motif of Ac93 dramatically reduced the interaction of Vps4 and Ac93 (28). To determine if the MIM1 motif plays an essential role in mediating the interaction, we made the deletion (MIM1 and dMIM1) and triple leucine-to-alanine substitution (L142/145/146A [3L/A1] and L149/152/154A [3L/A2]) constructs, together with the previously constructed L142/145A, L146A, L149A, and L152/154A (28) (Fig. 6E), to assess the effect on interaction. Y2H analysis showed that the positive interaction occurred only between MIM1 or L152/154A and Vta1 (Fig. 6F). BiFC assay revealed that the complemented fluorescent cells were observed in cells cotransfected with the plasmids expressing Nm-Vta1 and Cm-fused MIM1, L142/145A, L146A, L149A, L152/154A, or 3L/A1, but not found in cells expressing Nm-Vta1 and dMIM1-Cm or 3L/A2-Cm (Fig. 6G). Similar to that of wt Ac93 (42.7%), the percentage of fluorescent cells for MIM1 was 41.9%. Both were significantly higher than that for L152/154A (34.4%), L142/145A, L146A, L149A, and 3L/A1 (4.7%–6.1%) (Fig. 6H). In addition, MIM1 maintained the interaction with Vps4. However, dMIM1, 3L/A1, and 3L/A2 completely abolished the interaction (Fig. 6I and J). Combined with the prior data, these results indicated that the leucine residues within MIM1 of Ac93 play essential roles in mediating the interaction with the MIT domains of Vps4 and Vta1.

### Interaction of Vps4-Vta1 and Ac93 is required for efficient entry of BV

**Fig 7:**
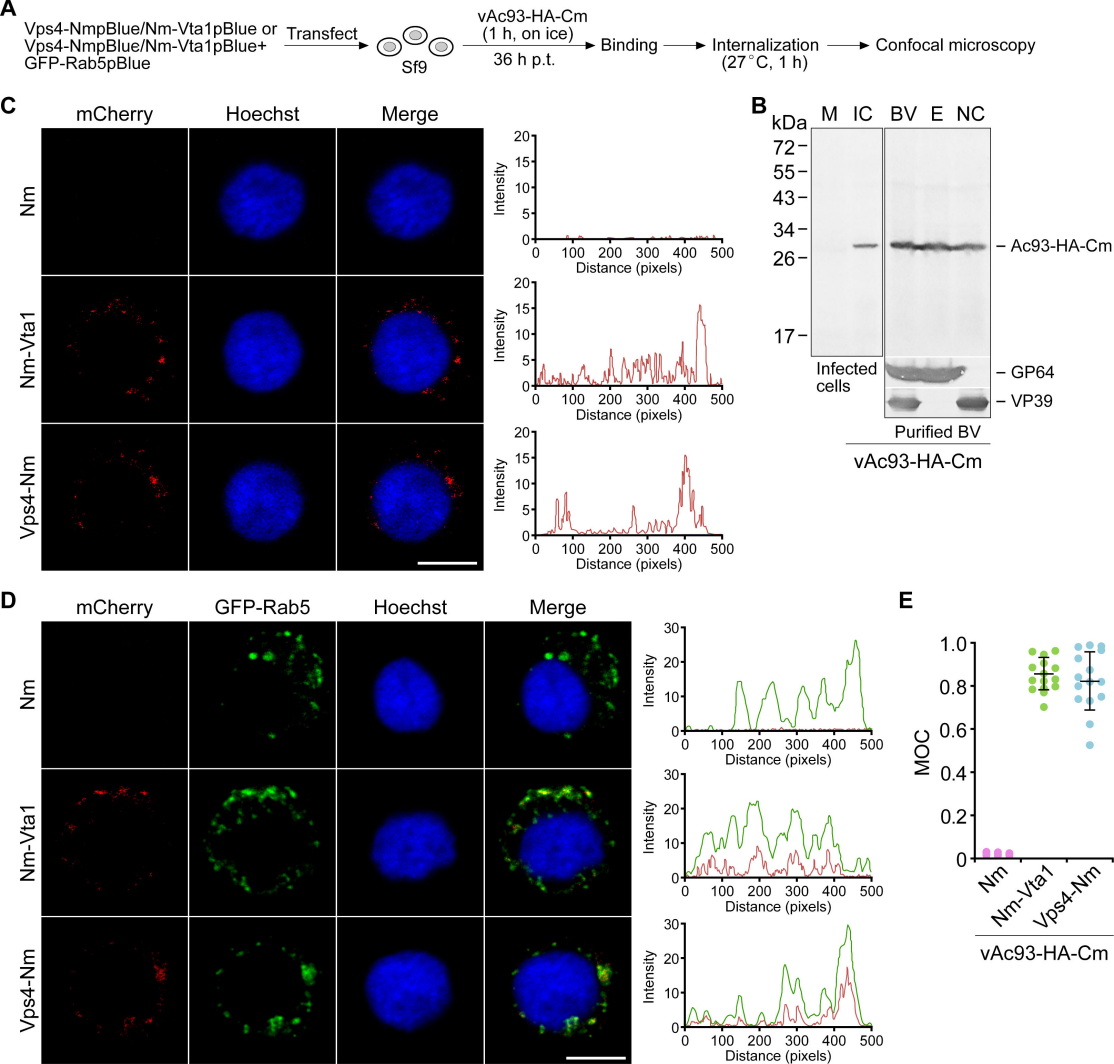
BiFC analysis of the interaction of Ac93 with Vps4 and Vta1 during BV entry. (A) Schematic representation of the assay. (B) Western blot analyses of the expression of Ac93-HA-Cm in infected Sf9 cells (72 h p.i.) and incorporation of it into BV and the fractions. BV, budded virus; E, envelope; IC, infected cells; M, mock-infected cells; NC, nucleocapsid. (C) Interaction assay in Sf9 cells transfected with the plasmid expressing Vps4-Nm or Nm-Vta1 and then inoculated with the virus vAc93-HA-Cm (MOI = 100). Plots of pixel intensity of the whole cell are shown on the right side of the images. (D and E) Interaction assay in Sf9 cells cotransfected with the plasmids expressing Vps4-Nm or Nm-Vta1 and GFP-Rab5 and then inoculated with the virus vAc93-HA-Cm (MOI = 100). Plots of pixel intensity of the whole cell are shown on the right side of the images. Colocalization of BiFC complemented mCherry fluorescence and GFP-Rab5 was evaluated by calculating MOC of 15 representative cells for each treatment (E). Scale bar, 10 µm.

Since Vps4-Vta1 and Ac93 adopt a similar interaction mode (MIM-MIT) as that of Vps4-Vta1 and ESCRT-III proteins, we wondered if the interactions are necessary for BV entry. To examine this, we first used BiFC to capture the potential interaction of Ac93 and Vps4 or Vta1 during BV entry (Fig. 7A). To perform this assay, a virus vAc93-HA-Cm was constructed by inserting Ac93-HA-Cm into an *ac93* knockout bacmid. Initial analysis indicated that Ac93-HA-Cm was expressed in infected Sf9 cells, assembled into the envelope and nucleocapsid of BV virions (Fig. 7B), and could efficiently rescue the virus infectivity (extended Fig. S6). Then, Sf9 cells were transfected with the plasmid NmpBlue, Nm-Vta1pBlue, or Vps4-NmpBlue, and at 36 h p.t., the cells were inoculated with vAc93-HA-Cm. Confocal microscopy revealed that, after a 1 h internalization period, the complemented BiFC fluorescence was detected in cells expressing Nm-Vta1 or Vps4-Nm but not in cells expressing Nm (Fig. 7C, the intensity plots). To verify if the detected fluorescence was localized in endosomes, Sf9 cells were cotransfected with the plasmids GFP-Rab5pBlue and NmpBlue, Nm-Vta1pBlue, or Vps4-NmpBlue, and at 36 h p.t., the cells were inoculated with vAc93-HA-Cm. Confocal microscopy indicated that the complemented mCherry fluorescence was colocalized with GFP-Rab5 (Fig. 7D, the intensity plots). MOC measurements showed that about 85.3% or 82.3% of the mCherry intensities in cells expressing Nm-Vta1 or Vps4-Nm were colocalized with that of GFP-Rab5 (Fig. 7E). These results suggested that Ac93 interacts with Vps4 and Vta1 in endosomes during BV entry.

**Fig 8:**
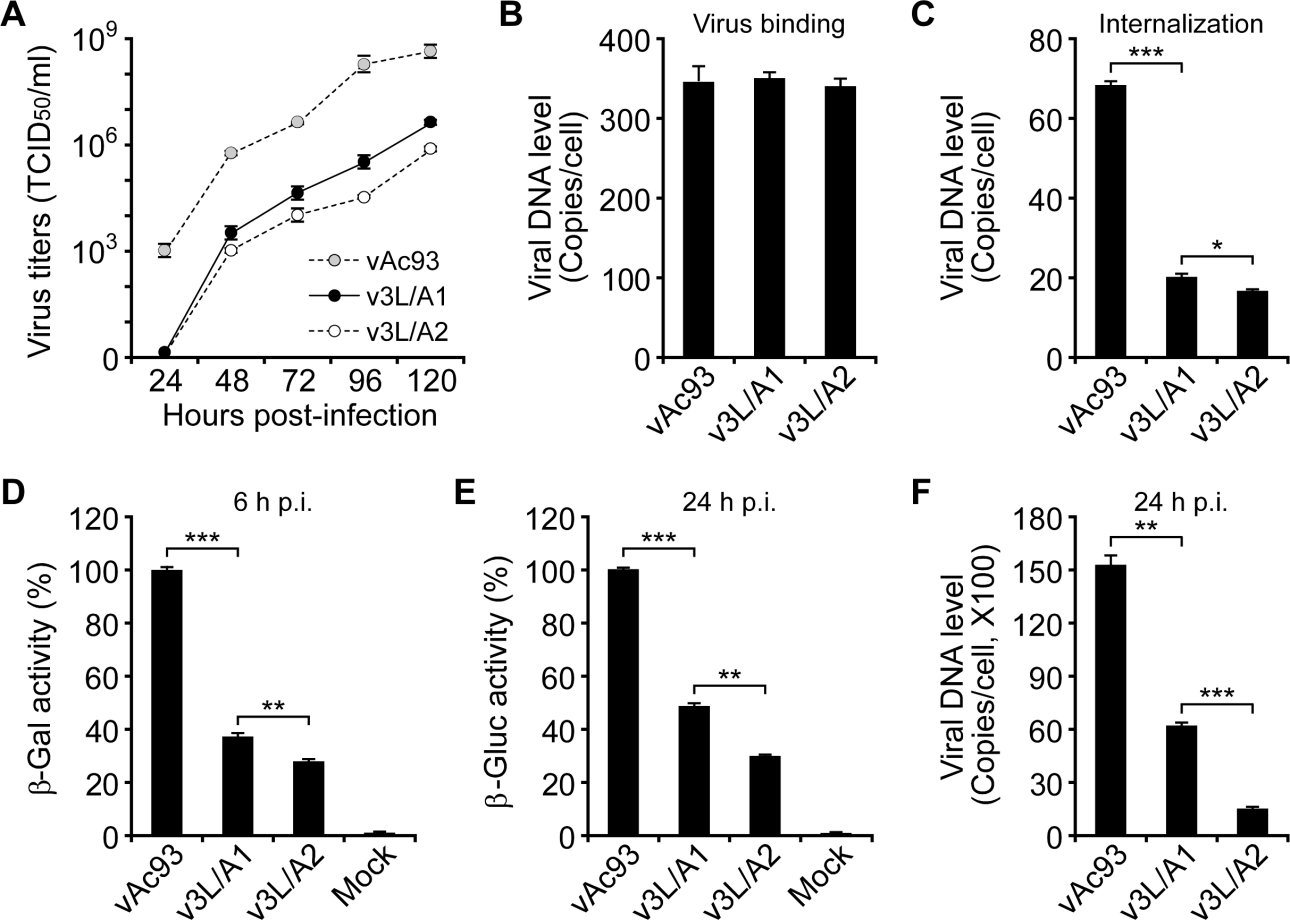
Effects of triple leucine-to-alanine substitutions in MIM1 of Ac93 on BV production and entry. (A) Multiple-step growth curves of wt or mutated Ac93 repaired *ac93*-knockout virus. Sf9 cells were infected with each of the viruses (MOI = 0.01), and the virus titers were measured at the indicated time points. (B and C) Virus binding and internalization assay. Sf9 cells were inoculated with each virus, and the binding and internalization efficiency were determined by measuring the viral genomic DNA using qPCR. (D–F) Analysis of the reporter genes’ expression and viral DNA replication. Following the procedure of viral internalization, the infected cells were lysed at 6 h and 24 h p.i. to measure the activities of β-Gal (D), β-Gluc (E), and the amount of viral DNA (F). Error bars represent SD of the mean of three replicates. *, *P* < 0.05; **, *P* < 0.01; ***, *P* < 0.001 (by paired two-tailed *t* test).

To determine the necessity of the interactions for BV entry, the *ac93* knockout virus was rescued by the MIM1-modified 3L/A1 or 3L/A2, and then used to assess the effects of disrupting the interaction on the virus binding and internalization. Similar to that observed for other leucine-to-alanine substitutions in MIM1 of Ac93 (28), the infectivities of v3L/A1 and v3L/A2 were significantly reduced at each time point (24–120 h p.i.) in comparison with that of wt Ac93 repaired virus (Fig. 8A). qPCR analysis indicated that mutation of MIM1 had no apparent effect on the virus binding (Fig. 8B). However, the amount of internalized v3L/A1 was remarkably decreased in comparison with that of vAc93, and a more severe reduction was detected for v3L/A2 (Fig. 8C). Consistent with the defect of virus internalization, at 6 h and 24 h p.i., the activities of reporters (β-Gal and β-Gluc) and the amount of viral DNA in Sf9 cells infected with v3L/A1 or v3L/A2 were also substantially reduced (Fig. 8D through F). Taken together, these results indicated that the interaction of Vps4-Vta1 and Ac93 is required for efficient entry of BV.

## DISCUSSION

The ESCRT system is essential for endosomal sorting and trafficking (1, 2). In recent years, the core components ESCRT-I/-II/-III and Vps4 are determined to be involved in the entry of diverse enveloped and non-enveloped DNA or RNA viruses (8–10, 13, 15–19). In addition, the ESCRT-0 component Hrs (Vps27 in yeast) is also necessary for entry of KSHV, LASV, LCMV, and RRV (8, 14, 19). Comparatively, ESCRT-I/-III and the Vps4-Vta1 complex but not ESCRT-0/-II are required for entry of AcMNPV. Since these viruses utilize distinct endocytic pathways to enter host cells, the available data suggest that, although the requirement of ESCRT-III/Vps4 for entry is common in viral infection, involvement of early-acting adaptors to recruit these core complexes substantially differs between AcMNPV and other viruses.

BV virions of AcMNPV enter host cells via CME (24). During trafficking within the endosomal system, membrane fusion and release of nucleocapsids into the cytoplasm may occur in early and maturing endosomes (30, 31). In yeast and mammalian cells, cargo sorting into ILVs begins in early endosomes. By binding to phosphatidylinositol 3-phosphate (PtdIns3P), ESCRT-0 targets to endosomes and clusters cargoes on the endosomal membrane. After recruiting ESCRT-I and ESCRT-II, they coordinate to create membrane invagination and transfer cargoes into nascent vesicles. Along with the membrane deformation, the ESCRT-III subunits are recruited by ESCRT-I/-II and polymerize to constrict the membrane neck (1). Even though the components of ESCRT-0 and ESCRT-II of *S. frugiperda* interact with other ESCRT proteins and share similar domain architectures as their homologs in yeast and mammals, depletion of these ESCRTs had no significant effect on virus production (Fig. 2D). In contrast, depletion or overexpression of dominant-negative (DN) ESCRT-I components Tsg101 and Vps28 does not affect BV internalization but impairs intracellular trafficking of the virus (12). Recent studies indicate that Bro1-domain proteins (ALIX and HD-PTP) recognize certain cargoes and directly recruit ESCRT-III, bypassing the need for ESCRT-0/-II in endosomal sorting (33). Additionally, several BV envelope proteins of AcMNPV, such as Ac75, Ac76, Ac78, Ac93, E18, E25, and P48, are identified to interact with ESCRT-III subunits (11). Thus, host Bro1 homologs and/or BV envelope proteins may serve as the early-acting adaptors to connect ESCRT-I/-III and promote the virus entry.

At the late stage of ILV formation, the monomeric or dimeric Vps4 and its cofactor Vta1 in the cytosol are recruited by ESCRT-III subunits to the membrane buds (1, 2), where Vps4 assembles to form an active hexamer that is stabilized by Vta1 dimer binding to adjacent subunits. Upon ATP hydrolysis, Vta1 promotes translocation of Vps4 hexamer along ESCRT-III filaments to drive membrane scission and disassemble ESCRT-III lattice (4, 5). In mammalian cells, depletion of LIP5 (Vta1 in yeast) does not affect the distribution and morphology of early endosomes but leads to accumulation of internalized epidermal growth factor receptor in intracellular vesicles (34). In Sf9 cells, depletion or overexpression of DN Vps4 results in defects of BV internalization and intracellular trafficking (10, 12). Similar defects were also observed in Vta1-depleted cells (Fig. 3C through G). It seems that depletion of Vps4 or Vta1 caused the internalized virions to be retained in early and maturing endosomes (Fig. 3H). In addition, although Vta1 interacts with Vps4 and they distribute as a similar pattern in infected cells, depletion of Vps4 but not Vta1 significantly reduces BV release. This correlates with their distinct levels at the plasma membrane. Surprisingly, depletion of Vta1 in BmN cells inhibits Bombyx mori nucleopolyhedrovirus (BmNPV) egress from MVBs (35), suggesting different baculoviruses may adopt distinct pathways for efficient budding. Together with prior and current results, our studies revealed that depletion of Vps4 or Vta1 may influence membrane fission and ESCRT-III filaments disassembly, which in turn impairs BV-specific ILV formation and trafficking and disrupts the virus entry.

During entry, BV virions encounter complicated pathways within the endosomes (31). In those scenarios, the viral envelope proteins may interplay with cellular factors, especially those of the ESCRT machinery, to facilitate sorting, trafficking, and release of nucleocapsids into the cytoplasm. To date, 14 proteins of AcMNPV, including Ac75, Ac76, Ac78, Ac93, E18, E25, E26, F-like, GP37, GP64, PIF4, P33, P48, and vUbi, are identified as the components of BV envelope (11, 20, 36), and four of them (Ac75, Ac93, P33, and P48) also associate with the nucleocapsid (37–40). Among them, the coding genes for Ac78, Ac93, E18, E25, PIF4, P33, and P48 are present in all sequenced baculovirus genomes (11). Deletion of E26, F-like, GP37, or PIF4 has no obvious negative effect on the virus infection in cell culture (41–44), while individual deletion of the others moderately or severely impairs infectious BV production or IM formation (37, 38, 40, 45–49). Except for knowing that GP64 has receptor-binding activity and mediates membrane fusion (25, 50), the requirement of other envelope proteins for BV entry remains unknown. Recently, these envelope proteins have been determined to form an interaction network, and eight of them (Ac75, Ac76, Ac78, Ac93, E18, E25, P48, and vUbi) interact with all subunits of ESCRT-III. However, the significance of these interactions in BV entry and/or egress is not clear (11). Current studies revealed that Ac75, E25, F-like, P33, P48, and vUbi weakly associate with Vps4 and Vta1. In contrast, Ac93 showed strong interaction with both proteins and played a central role in connecting the BV envelope proteins and Vps4-Vta1 (Fig. 5). These results highlight the possibility that BV envelope proteins cooperate with ESCRT-III and the Vps4-Vta1 complex to regulate the virus entry.

In the biogenesis of MVBs, a simple model is that ESCRT-III, Vps4, and Vta1 are sequentially recruited to the endosomal membrane (1). Initially, the membrane-localized ESCRT-III subunit Vps20 nucleates the formation of Snf7 filament. Then, the filament is capped and terminated by the Vps2-Vps24 subcomplex, which in turn recruits Ist1-Vps46. Finally, Ist1, Vps2, and Vps46 recruit the Vps4-Vta1-Vps60 complex via binding their C-terminal MIMs to MIT domains of Vps4 and Vta1 (51). Basically, two types of MIMs (MIM1 and MIM2) are identified in ESCRT-III proteins (52, 53). As known, Chm7 (orthologs in mammals termed charged MVB protein 7, CHMP7), Vps2 (CHMP2A/B), Vps24 (CHMP3), Vps46 (CHMP1A/B), and Vps60 (CHMP5) contain an MIM1 motif (D/E*xx*L*xx*RL*xx*L[K/R], *x* represents any other residue), while Snf7 (CHMP4A/B/C) and Vps20 (CHMP6) contain an MIM2 motif ([L/V]P*x*[V/L]P, *x* represents a hydrophilic residue), and Ist1 (CHMP8) contains one MIM1 and an MIM2 (2). Except for the MIM1 of Vps60/CHMP5, which has a high affinity to MIT2 of Vta1, the other MIM1 or MIM2 efficiently binds to MIT of Vps4 and MIT1 of Vta1 (53, 54). It seems that Vps60 acts as a negative regulator to control Vta1-mediated stimulation of Vps4 (55). Similar to ESCRT-III proteins, Ac93 is composed of five alpha helices and a C-terminal MIM1 motif (28). Deletion of MIM1 or mutation of the leucine residues within it severely abolished the interaction of Ac93 with the MIT domains of Vps4 and Vta1. Additionally, although Ac93 can associate with different domains of Vta1, it showed much stronger interaction with MIT1 than with MIT2 or the C-terminal region (without MIT domains) (Fig. 6). Intriguingly, in an internalization period, the complemented mCherry fluorescence of Ac93-Cm and Vps4-Nm or Nm-Vta1 was highly colocalized with GFP-Rab5, suggesting the interactions occurred in early and maturing endosomes. Consistently, disruption of the interaction severely impaired BV internalization (Fig. 8C). In addition, prior studies indicated that mutation of leucines in MIM1 of Ac93 interrupts egress of nucleocapsids from NE and impairs IMs formation (28). Together, these studies revealed that the coordination of Ac93 with the ESCRT system plays dual roles in regulating BV entry and egress.

**Fig 9:**
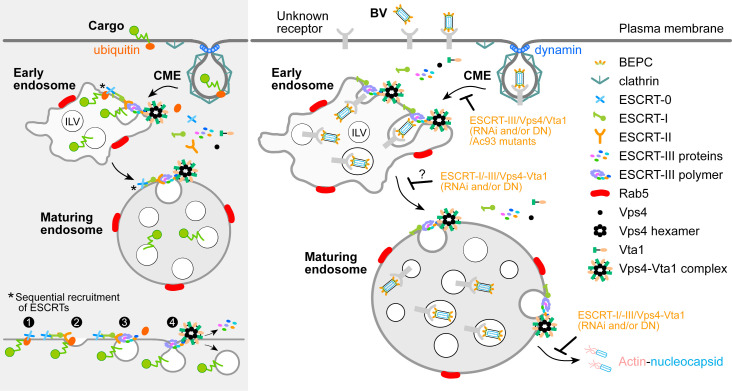
Models of ESCRT-mediated endosomal sorting, ILV formation, and BV virion entry. For endosomal sorting and ILV formation (the left shaded panel), a membrane ubiquitylated-cargo is internalized via CME and then recognized by ESCRT-0 on the endosomal membrane (step 1). Following sequential recruitment of ESCRT-I, ESCRT-II, and ESCRT-III, the cargo is sorted into the membrane bud (steps 2 to 3). As the bud neck is constricted by the ESCRT-III filament, Vps4 and its cofactor Vta1 are recruited to promote membrane scission and disassemble the ESCRT-III complex (step 4) (1, 2, 5). For BV entry (right panel), the viruses attach to the plasma membrane through the interaction of GP64 (maybe GP64 and other envelope proteins) with the unknown receptor(s) (50) and then enter host cells via CME (24). While transporting to and trafficking within the endosomal system, the BV envelope proteins complex may direct or cooperate with ESCRT-I to recruit ESCRT-III and the Vps4-Vta1 complex to generate BV-specific ILVs, which mediate the virus sorting and trafficking. After releasing from endosomes, the nucleocapsid is delivered by actin filaments to the nucleus (27). DN, dominant-negative.

In summary, we found that Vta1 but not ESCRT-0/-II was involved in the entry of AcMNPV. Seven BV envelope proteins, including the core or highly conserved Ac75, Ac93, E25, P33, and P48, were identified to interact with Vps4 and Vta1. Among them, Ac93 utilized its MIM1 motif to interact with the MIT domains of Vps4 and Vta1 and played a predominant role in connecting the viral proteins and Vps4-Vta1. Based on the prior and current studies, we proposed a model for the coordinated action of BV envelope proteins and the ESCRT system in facilitating entry of BV virions (Fig. 9, right panel). In this model, the viral proteins may direct or coordinate with ESCRT-I to recruit ESCRT-III and the Vps4-Vta1 complex to facilitate BV sorting and trafficking within endosomes. Since Ac75, Ac93, and P48 are also present on BV nucleocapsids and essential for IMs formation and nucleocapsids egress from NE (37, 38, 40), it is worth noting that the interactions of these viral proteins with Vps4-Vta1 may also be involved in those processes. The necessity and mechanism of the interactions need further investigation to clarify. Validation of this or other models may promote understanding of the ESCRT-dependent entry mechanisms of baculoviruses and other large DNA viruses.

## MATERIALS AND METHODS

### Cells and transfection

*S. frugiperda* cells (Sf9) were cultured at 27°C in Grace’s medium supplemented with 3.33 g/L lactalbumin hydrolysate, 3.33 g/L yeastolate, and 10% FBS (Thermo Fisher Scientific). Transfection of plasmid and viral bacmid DNAs and dsRNA in 6-well plates (1 × 10^6^ cells/well) or 12-well plates (2 × 10^5^ cells/well) was performed using a CaPO_4_ precipitation method (12).

### Mutagenesis and construction of plasmids and bacmids

Total RNA was isolated from Sf9 cells using the RNeasy Mini kit (QIAGEN) and reverse-transcribed into cDNA with the Evo M-MLV RT kit (Accurate Biology). The ORFs of the components of ESCRT-0/-II, Vta1 and its truncated forms dMIT1-2 (deletion of the N-terminal 156 aa), MIT1 (75 aa), MIT2 (93 aa), and MIT1-2 (168 aa) were amplified by PCR (primers listed in Table 1) using the cDNA as templates. dMIM1 (deletion of N139-L154), MIM1 (N139-L154), and substitutions (3L/A1 and 3L/A2) in MIM1 of Ac93 were generated by PCR using Ac93-HApBlue (28). PCR products were cloned into pMD18-T and verified by sequencing. Then, each fragment was isolated from the pMD18-T or from GFP-Tsg101pBlue, GFP-Vps28pBlue (12), L142/145A-CmpBlue, L146A-CmpBlue, L149A-CmpBlue, L152/154A-CmpBlue (28), Nm-dMIT1pBlue, Nm-dMIT2pBlue (29), or Vps4-gfppBlue (10) by using BamHI/EcoRI (HseI, Tsg101, Vps28, Vta1, and its truncations), BamHI/XhoI (Vps27), XbaI/EcoRI (Mutated Ac93, Vps4, Vps22, Vps25, and Vps36) and inserted into the BiFC vectors (HA-NmpBlue and Myc-CmpBlue), Y2H vectors (pGADT7 and pGBKT7), pIEnmCherry, or E231Q-mCherrypBlue (11). GFP-LaminBpBlue, GFP-Rab5pBlue, pIEmCherry, Vps4-gfppBlue, and the other BiFC and Y2H plasmids were used as previously described (10, 11, 28, 31).

**TABLE 1:** Primers for PCR and qPCR^*a*^

#Name	Sequence (5’ to 3’)	#Name	Sequence (5’ to 3’)
1. Hse1BF	ataggatccatggggattttcggcaattct	2. Hse1ER	atagaattctcagcatcgaggaggcgggggat
3. Vps27BF	ataggatccatgtttcgagctaataattttga	4. Vps27ER	atagaattcctcagtagtatcagag
5. Vps27EF	atagaattcccaaaaccttccacaac	6. Vps27XoR	atactcgagtcaatcgaaactgatcagctc
7. Vps22XF	aattctagaatgaggcgtcatgcaggagta	8. Vps22ER	aatgaattctgcagccacacactcggagaa
9. Vps25XF	aattctagaatggcagatatcgtatggccgt	10. Vps25ER	aatgaattcgaagaacttcactccctggtt
11. Vps36XF	aattctagaatggatagatttgaatatattgaagc	12. Vps36ER	aatgaattctgaggcttctcgtaaaaatag
13. Vta1BF	aatggatccatgtctgtgaatatacctg	14. MIT1ER	aatgaattcctattctcgatacgttttcttagct
15. MIT2F	aatggatccatgaatgaagcaattactaatg	16. MIT1-2R	aatgaattcctatgactgcattggtcctgg
17. dMIT1-2F	aatggatccatgaatggagagactcctgttc	18. Ac93XF	aattctagaatggcgactagcaaaacgatc
19. Ac93ER	atagaattcatttacaatttcaattccaatg	20. 3L/A1F	aatgcaagcgtcgcagcaaaacatttgcacaa
21. 3L/A1R	ttgtgcaaatgttttgctgcgacgcttgcatt	22. 3L/A2F	aacatgcacacaacgctaatgctattggaattg
23. 3L/A2R	caattccaatagcattagcgttgtgtgcatgtt	24. GP64pAR	ataaagcttcacactcgctatttggaac
25. 93M1F	ctagaatgaacgataatttaagtgtacttttgaaacatttgcacaacctaaatctcg		
26. 93M1R	aattcgagatttaggttgtgcaaatgtttcaaaagtacacttaaattatcgttcatt		
27. 93dMER	atagaattcgttgacgatctcgatgccgatttcccaattgacgggcacggcca		
28. HAEF	atagaattctacccatacgatgttccagattacgctgggacgtcgggtggaagcggt		
29. qHse1F	gctcactttcttagctggcg	30. qHse1R	ccaagtcacccgttacgaaat
31. qVps27F	gcagatggccgctaaactgca	32. qVps27R	taaccagcctggaactgttg
33. qVPS22F	agctgcggacgaggctcat	34. qVps22R	ccgcttgctttctgcaaca
35. qVps25F	ctctgtctgtaccctgttt	36. qVps25R	cttcactccctggttgtc
37. qVps36F	gatatacccaaggggcttgt	38. qVps36R	gtcttttacctggtaatgc
39. qVta1F	tactgtgactccagcaaccc	40. qVta1R	tcgtaagggacaaacccgc
41. ODVe56F	gatcttcctgcgggccaaacact	42. ODVe56R	aacaagaccgcgcctatcaacaaa
43. Hse1iF	ggatcctaatacgactcactatagggccgaactggtggaaaggata	44. Hse1iR	ggatcctaatacgactcactatagggcagcgagtggtacaggttca
45. Hse1F	ccgaactggtggaaaggata	46. Hse1R	cagcgagtggtacaggttca
47. Vps27iF1	ggatcctaatacgactcactatagggaggacgctgaggacaaagaa	48. Vps27iR1	ggatcctaatacgactcactatagggtgaactctttgcaatgccag
49. Vps27F1	aggacgctgaggacaaagaa	50. Vps27R1	tgaactctttgcaatgccag
51. Vps27iF2	ggatcctaatacgactcactatagggtctgtgagatgctccgtgac	52. Vps27iR2	ggatcctaatacgactcactatagggcagactcgcacctccttttc
53. Vps27F2	tctgtgagatgctccgtgac	54. Vps27R	cagactcgcacctccttttc
55. Vps22iF	ggatcctaatacgactcactatagggctcagtttaggcggcagttc	56. Vps22iR	ggatcctaatacgactcactatagggctttacccatcggcacaact
57. Vps22F	ctcagtttaggcggcagttc	58. Vps22R	ctttacccatcggcacaact
59. Vps25iF	ggatcctaatacgactcactatagggctcacacagaaacaagggca	60. Vps25iR	ggatcctaatacgactcactatagggctccataagctcgcatctcc
61. Vps25F	ctcacacagaaacaagggca	62. Vps25R	ctccataagctcgcatctcc
63. Vps36iF1	ggatcctaatacgactcactatagggaaagtggtggagtgtttggc	64. Vps36iR1	ggatcctaatacgactcactataggggcaaccatgtctttggcttt
65. Vps36F1	aaagtggtggagtgtttggc	66. Vps36R1	caaccatgtctttggcttt
67. Vps36iF2	ggatcctaatacgactcactataggggtactcatggattgtggggg	68. Vps36iR2	ggatcctaatacgactcactatagggtgccaggccttcaatagact
69. Vps36F2	tactcatggattgtggggg	70. Vps36R2	tgccaggccttcaatagact
71. Vps4iF	ggatcctaatacgactcactatagggggaaacacgaggcatcaact	72. Vps4iR	ggatcctaatacgactcactatagggaacttcctaagccggaccat
73. Vps4F	ggaaacacgaggcatcaact	74. Vps4R	aacttcctaagccggaccat
75. Vta1iF	ggatcctaatacgactcactatagggcacaagcccaccttgaaaat	76. Vta1iR	ggatcctaatacgactcactatagggcattaggatcgggcagaaaa
77. Vta1F	cacaagcccaccttgaaaat	78. Vta1R	cattaggatcgggcagaaaa
79. GFPiF	ggatcctaatacgactcactatagggacgtaaacggccacaagttc	80. GFPiR	ggatcctaatacgactcactatagggtgttctgctggtagtggtcg
81. GFPF	acgtaaacggccacaagttc	82. GFPR	tgttctgctggtagtggtcg
83. LtAiF	ggatcctaatacgactcactatagggcaccctctccacgaattg	84. LtAiR	ggatcctaatacgactcactatagggtagaagatgctgctgtttca
85. LtAF	caccctctccacgaattg	86. LtAR	tagaagatgctgctgtttca

^*a*^ The restriction enzyme sites and T7 promoter sequence were underlined. Primers 1–28 were used to amplify the ORF of the components of ESCRT-0/-II, Vta1, and its truncated forms, mutated Ac93, and the fragment HA-Cm-poly(A). Primers 29–42 were used for qPCR quantification of the transcripts of each gene and viral genomic DNA. Primers 43–86 were used for dsRNA synthesis.

To construct the bacmid, three transfer plasmids were first generated: (i) SK-GFPpBlue: a KpnI site was introduced at the SacI site of gfppBlue (10); (ii) GFP-dXpBlue: the XhoI site in gfppBlue was removed by inserting a synthesized fragment between HindIII and KpnI sites; (iii) Pac93-Ac93-HA-CmpBlue: the fragment HA-poly(A) between EcoRI and HindIII sites in Pac93-Ac93-HApBlue (28) was replaced by the PCR-amplified fragment HA-Cm-poly(A). Then, the ORF of β-Gal was amplified using LacZGUSpFB (31) and inserted into the XbaI/EcoRI sites of SK-GFPpBlue to replace the ORF of GFP and generate SK-LacZpBlue. Two cassettes containing the *ie1* promoter, *gfp* or *LacZ*, and the poly(A) of *gp64* (pA*gp64*) were isolated from GFP-dXpBlue (SacI/KpnI) and SK-LacZpBlue (KpnI/XhoI) and sequentially cloned into GFP-polyhedrinpFB (28) to replace the cassette encoding GFP and generate GFP-LacZpFB. To construct the bacmid expressing mCherry-tagged Vps4 or Vta1, a cassette containing the *ie1* promoter, Vps4-mCherry or mCherry-Vta1, and pA*gp64* was isolated from Vps4-mCherrypBlue or mCherry-Vta1pBlue using SacI/KpnI and inserted into GFP-LacZpFB. The resulting pFastBac plasmids were separately used to insert the target fragment into the *att*Tn7 site on an AcMNPV bacmid (bMON14272). mCherryBac was used as a control (11). To generate the modified Ac93 repaired bacmid, the ORF of 3L/A1 or 3L/A2 was isolated from the BiFC plasmid using XbaI/EcoRI and inserted into Pac93-Ac93-HApBlue to produce Pac93-3L/A1-HApBlue and Pac93-3L/A2-HApBlue. Then, the cassette containing the *ac93* promoter, Ac93-HA-Cm, 3L/A1-HA, or 3L/A2-HA, and pA*gp64* was isolated from Pac93-Ac93-HA-CmpBlue, Pac93-3L/A1-HApBlue, or Pac93-3L/A2-HApBlue using SacI/KpnI and inserted into GFP-LacZpFB to replace the cassette encoding GFP. Each of the donor plasmids was transformed into DH10B cells containing the *ac93-*knockout bacmid (bMON14272-*ac93*^ko^) (28) to generate bacmids of vAc93-HA-Cm, v3L/A1, and v3L/A2. The wt Ac93 repaired virus vAc93 was used as described (28).

### Transcription and RNAi assay

Sf9 cells in 6-well plates were infected with AcMNPV-LacZGUS (MOI = 5) (31). At 1–48 h p.i., total RNA was isolated using the RNeasy Mini kit (QIAGEN) and reverse-transcribed into cDNA with the Evo M-MLV RT kit (Accurate Biology). The transcripts of the components of ESCRT-0, -II, and *Vta1* were measured by qPCR. Each qPCR reaction mixture (20 µL) contained 10 µL SYBR Green Premix II (Accurate Biology), 2.5 µL each primer (2.5 µM) (Table 1), and 800 ng of the cDNA. A standard curve was generated by a serial dilution of the pGADT7 vector containing the ORF of each gene.

The dsRNA-based RNAi assay was performed as described earlier (12). PCR primers (Table 1) targeted to one or two fragments within the ORF of the components of ESCRT-0/-II or *Vta1* were designed with the SnapDragon tool (https://www.flyrnai.org/cgi-bin/RNAi_find_primers.pl). Primers of *gfp, LtA* (GenBank XM_006523731), and *Vps4* were used as described (12, 32). PCR products were transcribed into dsRNA with the T7 RiboMAX Express RNAi system (Promega). Then, Sf9 cells in 12-well plates were transfected with the dsRNA (5 µg/well for each gene, not mentioned elsewhere). At 48 h p.t., two sets of the cells were separately used to assess the cell viability with the CellTiter 96 AQ_ueous_ One Solution reagent (Promega) and measure the transcripts of each gene by qPCR, the other set of the cells was infected with AcMNPV-LacZGUS (MOI = 5). At 24 h p.i., the infectious BV was titered by a 50% tissue culture infection dose (TCID_50_) assay.

### Virus growth curve assay

Sf9 cells in 6-well plates were infected with (i) the virus vAc93, v3L/A1, or v3L/A2 (MOI = 0.01); (ii) the virus vAc93 or vAc93-HA-Cm (MOI = 5). After incubating at 27°C for 1 h, the viral inoculum was removed, and the cells were washed once with the medium. At 24–120 h p.i., infectious BV titers were measured by a TCID_50_ assay.

### BV purification and fraction

BV virions were purified and fractionated as described previously (56), with minor modifications. Briefly, Sf9 cells were infected with AcMNPV-3mC, AcMNPV-LacZGUS, vAc93, v3L/A1, v3L/A2, or vAc93-HA-Cm (MOI = 1). At 96 h p.i., BV virions were collected by centrifugation at 4°C, 28,000 rpm for 1 h (Himac CP100WX, P28S) and then loaded onto a 25%–56% (wt/vol) sucrose gradient and centrifuged again for 2 h. The virus band was removed and diluted in Grace’s medium and repelleted by centrifugation at 4°C, 28,000 rpm for 1 h (P40ST). After being resuspended in Grace’s medium and sterilized by filtration, the virus titer was determined by a TCID_50_ assay. Also, the viral genomic DNA was extracted using the DNeasy Blood & Tissue kit (QIAGEN) and quantified by qPCR amplification of a small fragment of ODV-e56 as previously described (10). As detected, one TCID_50_ is corresponding to about 50 copies of viral genomic DNA.

To fraction vAc93-HA-Cm into envelope and nucleocapsids, about 400 µg of purified viruses were lysed in 500 µL 1.0% NP-40 (10 mM Tris, pH 8.5) for 30 min, and then layered onto a 30%–70% (wt/vol) glycerol gradient and centrifuged at 4°C, 28,000 rpm for 1 h (P40ST). The envelope and nucleocapsid fractions were dialyzed against 10 mM Tris (pH 7.4) and concentrated using the Amicon Ultra centrifugal filter (3 kDa MWCO, Merck).

### Virus binding and internalization assay

Sf9 cells in 12-well plates were transfected with the dsRNA of *gfp*, *Vps4*, or *Vta1*. At 48 h p.t., the cells were chilled at 4°C for 30 min and then inoculated with the purified virus AcMNPV-LacZGUS (MOI = 10) on ice for 1 h. After removing the inoculum and washing the cells once with Grace’s medium, one set of the cells was lysed to extract DNA using the DNeasy Blood & Tissue kit (QIAGEN), and another set of the cells was incubated at 27°C for 1 h. Then, the bound viruses were inactivated and removed by treating the cells with the citrate buffer (10 mM KCl, 135 mM NaCl, 40 mM sodium citrate, pH 3.1) for 1 min (31) and then the cells were lysed to extract DNA. Viral genomic DNA was measured by qPCR as described above. Following the same procedure, Sf9 cells in 12-well plates were chilled and inoculated with the purified virus vAc93, v3L/A1, or v3L/A2 (MOI = 10) to assess the virus binding and internalization.

### Reporter gene expression and viral DNA replication assay

Sf9 cells in 12-well plates were transfected with the dsRNA of *gfp*, *Vps4*, or *Vta1*. At 48 h p.t., the cells were infected with the purified virus AcMNPV-LacZGUS (MOI = 5). At 6 h p.i., one set of the cells was lysed using 1% Triton X-100 containing 2 mg/mL Chlorophenol red-β-D-galactopyranoside (Roche Diagnostics GmbH) to measure the activity of β-Gal. At 24 h p.i., one set of the cells was lysed using 1% Triton X-100 containing 1 mM 4-nitrophenyl β-D-glucuronide (Merck) to measure the activity of β-Gluc. Another set of the cells was lysed to extract DNA and measure the viral genomic DNA by qPCR. Similarly, Sf9 cells in 12-well plates were infected with the purified virus vAc93, v3L/A1, or v3L/A2 (MOI = 5), and at 6 h and 24 h p.i., the activities of β-Gal, β-Gluc, and the amount of viral DNA were measured.

### Virus release assay

Sf9 cells in 12-well plates were transfected with the dsRNA of *gfp*, *Vps4*, or *Vta1*. At 48 h p.t., the cells were transfected again with the bacmid DNA of AcMNPV-LacZGUS (4 µg/well). After transfecting the viral DNA for 24 h, infectious BVs were titered, and the cells were lysed to measure the activities of β-Gal and β-Gluc.

### Y2H and BiFC

Y2H was assayed with the Matchmaker Gold Yeast Two-Hybrid System (Clontech). The competent cells of Y2H Gold were transformed with a pair of plasmids separately encoding the components of ESCRT-0/-I/-II/-III, Vps4, Vta1, or BV envelope proteins. The cells were grown at 30°C on double droplet plates (SD/-Leu/-Trp) for 3 days. Single colonies were cultured in liquid YPD medium overnight. Then, the cells were diluted to plate on the selective medium (SD/-Ade/-His/-Leu/-Trp) containing Aureobasidin A (70 ng/mL) and incubated at 30°C for 3–5 days.

For BiFC, Sf9 cells in 12-well plates were transfected with a pair of plasmids (2 µg/well for each plasmid). At 36 h p.t., the expression of each protein was detected by western blotting, and the complemented mCherry fluorescence was analyzed by epifluorescence microscopy. The interaction of paired proteins was estimated by the percentage of fluorescent cells in each field as described previously (12).

### Confocal microscopy

Sf9 cells were seeded on glass coverslips in 12-well plates. For cellular localization assay, the cells were cotransfected with (i) GFP-LaminBpBlue (0.3 µg/well) and pIEmCherry, mCherry-Vta1pBlue, or Vps4-mCherrypBlue (2 µg/well); (ii) mCherry-Vta1pBlue and gfppBlue or Vps4-gfppBlue (2 µg/well for each plasmid); (iii) GFP-LaminBpBlue (0.3 µg/well) and the bacmid expressing mCherry, mCherry-Vta1, or Vps4-mCherry (4 µg/well). At 36 h p.t. (for plasmid transfection) or 36 h and 72 h p.t. (for bacmid transfection), the cells were fixed with 3.7% paraformaldehyde for 30 min and then permeabilized with 0.05% Triton X-100 for 1 min. The cell nuclei were stained with Hoechst 33258 (Thermo Fisher Scientific). Images were scanned in sequential mode using a Leica TCS SP8 confocal microscope (Leica Microsystems Inc.) with a 63× objective (oil immersion, NA1.4).

For colocalization assay, Sf9 cells were transfected with the bacmid expressing mCherry, mCherry-Vta1, or Vps4-mCherry (4 µg/well). At 72 h p.t., the cells were fixed with 3.7% paraformaldehyde and blocked with 1% gelatin. Then, the cells were incubated with the anti-GP64 MAb AcV1 (Santa Cruz Biotechnology) and the Alexa Fluor 488-conjugated goat anti-mouse IgG (H+L) (Thermo Fisher Scientific) as previously described (11). For the virus entry assay, the cells were transfected or cotransfected with (i) the dsRNA of *gfp*, *Vps4*, or *Vta1*; (ii) GFP-Rab5pBlue (0.5 µg/well) and the dsRNA of *LtA*, *Vps4*, or *Vta1*; (iii) Nm-HApBlue, Nm-HA-Vta1pBlue, or Vps4-HA-NmpBlue (2 µg/well for each plasmid); (iv) GFP-Rab5pBlue (0.5 µg/well) and Nm-HApBlue, Nm-HA-Vta1pBlue, or Vps4-HA-NmpBlue (2 µg/well for each plasmid). At 36 h p.t. (plasmid transfection) or 48 h p.t. (dsRNA transfection), following the procedure for virus internalization assay, the cells were inoculated with the purified virus vAc93-HA-Cm (MOI = 100) or AcMNPV-3mC (MOI = 20). Then, the cells were fixed and stained with Hoechst 33258. Images were analyzed with the Fiji image processing package (57).

### Western blotting

The transfected or infected Sf9 cells, the purified BV and its envelope and nucleocapsid fractions of vAc93-HA-Cm were lysed with RIPA buffer containing the complete protease inhibitor cocktail (Roche Diagnostics GmbH) and separated on 10%–15% SDS-PAGE gels. Then, the proteins were transferred to PVDF membrane (0.45 µm, GVS) and detected using the MAb anti-HA 12CA5 (Bioss), anti-Myc 2G8D5 (Genscript), anti-GP64 AcV5 (Santa Cruz Biotechnology), or the pAb anti-VP39 (36) and the alkaline phosphatase-conjugated goat anti-mouse or anti-rabbit IgG (H+L) secondary antibody (Promega) and the BCIP/NBT color development kit (Coolaber).

## Data Availability

Extended data are available at https://doi.org/10.5061/dryad.g1jwstr2k and https://doi.org/10.5281/zenodo.14908530.

## References

[1] Vietri M, Radulovic M, Stenmark H. 2020. The many functions of ESCRTs. Nat Rev Mol Cell Biol 21:25–42. doi:10.1038/s41580-019-0177-4 31705132

[2] Schöneberg J, Lee I-H, Iwasa JH, Hurley JH. 2017. Reverse-topology membrane scission by the ESCRT proteins. Nat Rev Mol Cell Biol 18:5–17. doi:10.1038/nrm.2016.121 27703243 PMC5198518

[3] McCullough J, Frost A, Sundquist WI. 2018. Structures, functions, and dynamics of ESCRT-III/Vps4 membrane remodeling and fission complexes. Annu Rev Cell Dev Biol 34:85–109. doi:10.1146/annurev-cellbio-100616-060600 30095293 PMC6241870

[4] Xiao J, Xia H, Zhou J, Azmi IF, Davies BA, Katzmann DJ, Xu Z. 2008. Structural basis of Vta1 function in the multivesicular body sorting pathway. Dev Cell 14:37–49. doi:10.1016/j.devcel.2007.10.013 18194651 PMC2775496

[5] Monroe N, Han H, Shen PS, Sundquist WI, Hill CP. 2017. Structural basis of protein translocation by the Vps4-Vta1 AAA ATPase. Elife 6:e24487. doi:10.7554/eLife.24487 28379137 PMC5413351

[6] Rivera-Cuevas Y, Carruthers VB. 2023. The multifaceted interactions between pathogens and host ESCRT machinery. PLoS Pathog 19:e1011344. doi:10.1371/journal.ppat.1011344 37141275 PMC10159163

[7] Lippincott-Schwartz J, Freed EO, van Engelenburg SB. 2017. A consensus view of ESCRT-mediated human immunodeficiency virus type 1 abscission. Annu Rev Virol 4:309–325. doi:10.1146/annurev-virology-101416-041840 28715971 PMC6941200

[8] Pasqual G, Rojek JM, Masin M, Chatton JY, Kunz S. 2011. Old world arenaviruses enter the host cell via the multivesicular body and depend on the endosomal sorting complex required for transport. PLoS Pathog 7:e1002232. doi:10.1371/journal.ppat.1002232 21931550 PMC3169553

[9] Chen X, Liang Y, Weng Z, Hu C, Peng Y, Sun Y, Gao Q, Huang Z, Tang S, Gong L, Zhang G. 2024. ALIX and TSG101 are essential for cellular entry and replication of two porcine alphacoronaviruses. PLoS Pathog 20:e1012103. doi:10.1371/journal.ppat.1012103 38489378 PMC10971774

[10] Li Z, Blissard GW. 2012. Cellular VPS4 is required for efficient entry and egress of budded virions of Autographa californica multiple nucleopolyhedrovirus. J Virol 86:459–472. doi:10.1128/JVI.06049-11 22072775 PMC3255925

[11] Bai L, Sun Y, Yue X, Ji N, Yan F, Yang T, Feng G, Guo Y, Li Z. 2024. Multifaceted interactions between host ESCRT-III and budded virus-related proteins involved in entry and egress of the baculovirus Autographa californica multiple nucleopolyhedrovirus. J Virol 98:e0190023. doi:10.1128/jvi.01900-23 38289107 PMC10878073

[12] Yue Q, Yu Q, Yang Q, Xu Y, Guo Y, Blissard GW, Li Z. 2018. Distinct roles of cellular ESCRT-I and ESCRT-III proteins in efficient entry and egress of budded virions of Autographa californica multiple nucleopolyhedrovirus. J Virol 92:e01636-17. doi:10.1128/JVI.01636-17 29046462 PMC5730794

[13] Liu C, Liu Y, Zhou J, Chen X, Chen H, Hu J, Chen J, Zhang J, Sun R, Wei J, Go YY, Morita E, Zhou B. 2022. Cellular ESCRT components are recruited to regulate the endocytic trafficking and RNA replication compartment assembly during classical swine fever virus infection. PLoS Pathog 18:e1010294. doi:10.1371/journal.ppat.1010294 35120190 PMC8849529

[14] Veettil MV, Kumar B, Ansari MA, Dutta D, Iqbal J, Gjyshi O, Bottero V, Chandran B. 2016. ESCRT-0 component hrs promotes macropinocytosis of kaposi’s sarcoma-associated herpesvirus in human dermal microvascular endothelial cells. J Virol 90:3860–3872. doi:10.1128/JVI.02704-15 26819309 PMC4810545

[15] Kumar B, Roy A, Veettil MV, Chandran B. 2018. Insight into the roles of E3 ubiquitin ligase c-Cbl, ESCRT machinery, and host cell signaling in kaposi’s sarcoma-associated herpesvirus entry and trafficking. J Virol 92:e01376-17. doi:10.1128/JVI.01376-17 29167336 PMC5790950

[16] Shtanko O, Nikitina RA, Altuntas CZ, Chepurnov AA, Davey RA. 2014. Crimean-Congo hemorrhagic fever virus entry into host cells occurs through the multivesicular body and requires ESCRT regulators. PLoS Pathog 10:e1004390. doi:10.1371/journal.ppat.1004390 25233119 PMC4169490

[17] Broniarczyk J, Pim D, Massimi P, Bergant M, Goździcka-Józefiak A, Crump C, Banks L. 2017. The VPS4 component of the ESCRT machinery plays an essential role in HPV infectious entry and capsid disassembly. Sci Rep 7:45159. doi:10.1038/srep45159 28349933 PMC5368633

[18] Karjalainen M, Rintanen N, Lehkonen M, Kallio K, Mäki A, Hellström K, Siljamäki V, Upla P, Marjomäki V. 2011. Echovirus 1 infection depends on biogenesis of novel multivesicular bodies. Cell Microbiol 13:1975–1995. doi:10.1111/j.1462-5822.2011.01685.x 21899700

[19] Silva-Ayala D, López T, Gutiérrez M, Perrimon N, López S, Arias CF. 2013. Genome-wide RNAi screen reveals a role for the ESCRT complex in rotavirus cell entry. Proc Natl Acad Sci U S A 110:10270–10275. doi:10.1073/pnas.1304932110 23733942 PMC3690850

[20] Blissard GW, Theilmann DA. 2018. Baculovirus Entry and Egress from Insect Cells. Annu Rev Virol 5:113–139. doi:10.1146/annurev-virology-092917-043356 30004832

[21] van Oers MM, Pijlman GP, Vlak JM. 2015. Thirty years of baculovirus-insect cell protein expression: from dark horse to mainstream technology. J Gen Virol 96:6–23. doi:10.1099/vir.0.067108-0 25246703

[22] Possee RD, Chambers AC, Graves LP, Aksular M, King LA. 2020. Recent developments in the use of baculovirus expression vectors. Curr Issues Mol Biol 34:215–230. doi:10.21775/cimb.034.215 31167962

[23] Tsai CH, Wei SC, Lo HR, Chao YC. 2020. Baculovirus as versatile vectors for protein display and biotechnological applications. Curr Issues Mol Biol 34:231–256. doi:10.21775/cimb.034.231 31167963

[24] Long G, Pan X, Kormelink R, Vlak JM. 2006. Functional entry of baculovirus into insect and mammalian cells is dependent on clathrin-mediated endocytosis. J Virol 80:8830–8833. doi:10.1128/JVI.00880-06 16912330 PMC1563848

[25] Blissard GW, Wenz JR. 1992. Baculovirus gp64 envelope glycoprotein is sufficient to mediate pH-dependent membrane fusion. J Virol 66:6829–6835. doi:10.1128/JVI.66.11.6829-6835.1992 1404622 PMC240187

[26] Guo J, Li S, Bai L, Zhao H, Shang W, Zhong Z, Maimaiti T, Gao X, Ji N, Chao Y, Li Z, Du D. 2024. Structural transition of GP64 triggered by a pH-sensitive multi-histidine switch. Nat Commun 15:7668. 39227374 10.1038/s41467-024-51799-4 PMC11372198

[27] Ohkawa T, Volkman LE, Welch MD. 2010. Actin-based motility drives baculovirus transit to the nucleus and cell surface. J Cell Biol 190:187–195. doi:10.1083/jcb.201001162 20660627 PMC2930276

[28] Liu T, Li Y, Qiao B, Jiang Y, Ji N, Li Z. 2020. Disrupting the association of Autographa californica multiple nucleopolyhedrovirus Ac93 with cellular ESCRT-III/Vps4 hinders nuclear egress of nucleocapsids and intranuclear microvesicles formation. Virology (Auckl) 541:85–100. doi:10.1016/j.virol.2019.12.003 32056718

[29] Sun Y, Li Y, Wang S, Yu Q, Yue Q, Li Z. 2019. Effects of deletion of MIT domains of host Vta1 on replication of Autographa californica multiple nucleopolyhedrovirus. Acta Microbiol Sinica 59:247–257.

[30] Qin F, Xu C, Hu J, Lei C, Zheng Z, Peng K, Wang H, Sun X. 2019. Dissecting the cell entry pathway of baculovirus by single-particle tracking and quantitative electron microscopic analysis. J Virol 93:e00033-19. doi:10.1128/JVI.00033-19 30760565 PMC6450101

[31] Yue Q, Li J, Guo Y, Yan F, Liu X, Blissard GW, Li Z. 2020. Efficient entry of budded virions of Autographa californica multiple nucleopolyhedrovirus into spodoptera frugiperda cells is dependent on dynamin, Rab5, and Rab11. Insect Biochem Mol Biol 123:103409. doi:10.1016/j.ibmb.2020.103409 32417416

[32] Wang X-X, Zhang Y, Zhang Z-F, Tian H-G, Liu T-X. 2016. Deciphering the function of octopaminergic signaling on wing polyphenism of the pea aphid acyrthosiphon pisum. Front Physiol 7:603. doi:10.3389/fphys.2016.00603 28018234 PMC5145873

[33] Pashkova N, Gakhar L, Winistorfer SC, Sunshine AB, Rich M, Dunham MJ, Yu L, Piper RC. 2013. The yeast alix homolog Bro1 functions as a ubiquitin receptor for protein sorting into multivesicular endosomes. Dev Cell 25:520–533. doi:10.1016/j.devcel.2013.04.007 23726974 PMC3755756

[34] Ward DM, Vaughn MB, Shiflett SL, White PL, Pollock AL, Hill J, Schnegelberger R, Sundquist WI, Kaplan J. 2005. The Role of LIP5 and CHMP5 in multivesicular body formation and HIV-1 budding in mammalian cells. Journal of Biological Chemistry 280:10548–10555. doi:10.1074/jbc.M413734200 15644320

[35] Yao C, Pan S, Xu Y, Lu M, Zhao Y, Huo J, Hao B, Huang J. 2023. Bombyx mori nucleopolyhedrovirus hijacks multivesicular body as an alternative envelopment platform for budded virus egress. J Virol 97:e0004123. doi:10.1128/jvi.00041-23 36916914 PMC10062136

[36] Wang R, Deng F, Hou D, Zhao Y, Guo L, Wang H, Hu Z. 2010. Proteomics of the Autographa californica nucleopolyhedrovirus budded virions. J Virol 84:7233–7242. doi:10.1128/JVI.00040-10 20444894 PMC2898249

[37] Shi A, Hu Z, Zuo Y, Wang Y, Wu W, Yuan M, Yang K. 2018. Autographa californica multiple nucleopolyhedrovirus ac75 Is required for the nuclear egress of nucleocapsids and intranuclear microvesicle formation. J Virol 92:e01509-17. doi:10.1128/JVI.01509-17 29212928 PMC5790941

[38] Yuan M, Huang Z, Wei D, Hu Z, Yang K, Pang Y. 2011. Identification of Autographa californica nucleopolyhedrovirus ac93 as a core gene and its requirement for intranuclear microvesicle formation and nuclear egress of nucleocapsids. J Virol 85:11664–11674. doi:10.1128/JVI.05275-11 21880748 PMC3209287

[39] Nie Y, Fang M, Theilmann DA. 2011. Autographa californica multiple nucleopolyhedrovirus core gene ac92 (p33) is required for efficient budded virus production. Virology (Auckl) 409:38–45. doi:10.1016/j.virol.2010.09.023 20965540

[40] Wang Y, Cai Q, Chen J, Huang Z, Wu W, Yuan M, Yang K. 2019. Autographa californica multiple nucleopolyhedrovirus P48 (Ac103) is required for the efficient formation of virus-induced intranuclear microvesicles. Virol Sin 34:712–721. doi:10.1007/s12250-019-00147-8 31292829 PMC6889236

[41] Nie Y, Fang M, Theilmann DA. 2009. AcMNPV AC16 (DA26, BV/ODV-E26) regulates the levels of IE0 and IE1 and binds to both proteins via a domain located within the acidic transcriptional activation domain. Virology (Auckl) 385:484–495. doi:10.1016/j.virol.2008.12.020 19150105

[42] Lung OY, Cruz-Alvarez M, Blissard GW. 2003. Ac23, an envelope fusion protein homolog in the baculovirus Autographa californica multicapsid nucleopolyhedrovirus, is a viral pathogenicity factor. J Virol 77:328–339. doi:10.1128/jvi.77.1.328-339.2003 12477838 PMC140606

[43] Cheng XW, Krell PJ, Arif BM. 2001. P34.8 (GP37) is not essential for baculovirus replication. J Gen Virol 82:299–305. doi:10.1099/0022-1317-82-2-299 11161266

[44] Fang M, Nie Y, Harris S, Erlandson MA, Theilmann DA. 2009. Autographa californica multiple nucleopolyhedrovirus core gene ac96 encodes a per Os infectivity factor (PIF-4). J Virol 83:12569–12578. doi:10.1128/JVI.01141-09 19759145 PMC2786767

[45] Wu W, Passarelli AL. 2010. Autographa californica multiple nucleopolyhedrovirus Ac92 (ORF92, P33) is required for budded virus production and multiply enveloped occlusion-derived virus formation. J Virol 84:12351–12361. doi:10.1128/JVI.01598-10 20861245 PMC2976406

[46] Hu Z, Yuan M, Wu W, Liu C, Yang K, Pang Y. 2010. Autographa californica multiple nucleopolyhedrovirus ac76 is involved in intranuclear microvesicle formation. J Virol 84:7437–7447. doi:10.1128/JVI.02103-09 20484514 PMC2897645

[47] Tao XY, Choi JY, Kim WJ, Lee JH, Liu Q, Kim SE, An SB, Lee SH, Woo SD, Jin BR, Je YH. 2013. The Autographa californica multiple nucleopolyhedrovirus ORF78 is essential for budded virus production and general occlusion body formation. J Virol 87:8441–8450. doi:10.1128/JVI.01290-13 23698311 PMC3719795

[48] Chen L, Hu X, Xiang X, Yu S, Yang R, Wu X. 2012. Autographa californica multiple nucleopolyhedrovirus odv-e25 (Ac94) is required for budded virus infectivity and occlusion-derived virus formation. Arch Virol 157:617–625. doi:10.1007/s00705-011-1211-9 22218963

[49] McCarthy CB, Theilmann DA. 2008. AcMNPV ac143 (odv-e18) is essential for mediating budded virus production and is the 30th baculovirus core gene. Virology (Auckl) 375:277–291. doi:10.1016/j.virol.2008.01.039 18328526

[50] Zhou J, Blissard GW. 2008. Identification of a GP64 subdomain involved in receptor binding by budded virions of the baculovirus Autographica californica multicapsid nucleopolyhedrovirus. J Virol 82:4449–4460. doi:10.1128/JVI.02490-07 18287233 PMC2293031

[51] Rue SM, Mattei S, Saksena S, Emr SD. 2008. Novel Ist1-Did2 complex functions at a late step in multivesicular body sorting. Mol Biol Cell 19:475–484. doi:10.1091/mbc.e07-07-0694 18032584 PMC2230597

[52] Obita T, Saksena S, Ghazi-Tabatabai S, Gill DJ, Perisic O, Emr SD, Williams RL. 2007. Structural basis for selective recognition of ESCRT-III by the AAA ATPase Vps4. Nature New Biol 449:735–739. doi:10.1038/nature06171 17928861

[53] Yang Z, Vild C, Ju J, Zhang X, Liu J, Shen J, Zhao B, Lan W, Gong F, Liu M, Cao C, Xu Z. 2012. Structural basis of molecular recognition between ESCRT-III-like protein Vps60 and AAA-ATPase regulator Vta1 in the multivesicular body pathway. J Biol Chem 287:43899–43908. doi:10.1074/jbc.M112.390724 23105107 PMC3527972

[54] Shim S, Merrill SA, Hanson PI. 2008. Novel interactions of ESCRT-III with LIP5 and VPS4 and their implications for ESCRT-III disassembly. Mol Biol Cell 19:2661–2672. doi:10.1091/mbc.e07-12-1263 18385515 PMC2397308

[55] Vild CJ, Li Y, Guo EZ, Liu Y, Xu Z. 2015. A novel mechanism of regulating the ATPase VPS4 by its cofactor LIP5 and the endosomal sorting complex required for transport (ESCRT)-III protein CHMP5. J Biol Chem 290:7291–7303. doi:10.1074/jbc.M114.616730 25637630 PMC4358147

[56] Braunagel SC, Summers MD. 1994. Autographa californica nuclear polyhedrosis virus, PDV, and ECV viral envelopes and nucleocapsids: structural proteins, antigens, lipid and fatty acid profiles. Virology (Auckl) 202:315–328. doi:10.1006/viro.1994.1348 8009843

[57] Schindelin J, Arganda-Carreras I, Frise E, Kaynig V, Longair M, Pietzsch T, Preibisch S, Rueden C, Saalfeld S, Schmid B, Tinevez JY, White DJ, Hartenstein V, Eliceiri K, Tomancak P, Cardona A. 2012. Fiji: an open-source platform for biological-image analysis. Nat Methods 9:676–682. doi:10.1038/nmeth.2019 22743772 PMC3855844

